# Spatial Stochastic Model of the Pre-B Cell Receptor

**DOI:** 10.1109/TCBB.2022.3166149

**Published:** 2023-02-03

**Authors:** Romica Kerketta, M. Frank Erasmus, Bridget S. Wilson, Ádám M. Halász, Jeremy S. Edwards

**Affiliations:** AbbVie, Inc., Chicago, IL 60064 USA.; Specifica, Inc., Santa Fe, NM 87501 USA.; Department of Pathology and the Comprehensive Cancer Center, University of New Mexico Health Sciences Center, Albuquerque, NM 87102 USA.; Department of Mathematics, West Virginia University, Morgantown, WV 26506 USA.; Department of Chemistry and Chemical Biology, University of New Mexico, Albuquerque, Albuquerque, NM 87131 USA, and also with the Comprehensive Cancer Center, University of New Mexico Health Sciences Center, Albuquerque, NM 87102 USA.

**Keywords:** Agent based models, cell signaling, rule-based models, stochastic simulation methods

## Abstract

Survival and proliferation of immature B lymphocytes requires expression and tonic signaling of the pre-B cell receptor (pre-BCR). This low level, ligand-independent signaling is likely achieved through frequent, but short-lived, homo interactions. Tonic signaling is also central in the pathology of precursor B acute lymphoblastic leukemia (B-ALL). In order to understand how repeated, transient events can lead to sustained signaling and to assess the impact of receptor accumulation induced by the membrane landscape, we developed a spatial stochastic model of receptor aggregation and downstream signaling events. Our rule- and agent-based model builds on previous mature BCR signaling models and incorporates novel parameters derived from single particle tracking of pre-BCR on surfaces of two different B-ALL cell lines, 697 and Nalm6. Live cell tracking of receptors on the two cell lines revealed characteristic differences in their dimer dissociation rates and diffusion coefficients. We report here that these differences affect pre-BCR aggregation and consequent signal initiation events. Receptors on Nalm6 cells, which have a lower off-rate and lower diffusion coefficient, more frequently form higher order oligomers than pre-BCR on 697 cells, resulting in higher levels of downstream phosphorylation in the Nalm6 cell line.

## 1 Introduction

THE precursor B cell receptor (pre-BCR) appears early in the developmental pathway of B lymphocytes and serves as a checkpoint for the progression of B cell progenitors into mature B lymphocytes [1]. Expressed exclusively on the surface of precursor B cells, it is composed of a re-arranged immunoglobulin heavy chain (IgH) complexed with the *λ*5 and VpreB components of the surrogate light chain (SLC) [1]. The pre-BCR is also non-covalently attached to Ig*α* (CD79a) and Ig*β* (CD79b), heterodimeric subunits containing immunoreceptor tyrosine-based activation motifs (ITAMs) that are critical to propagate signaling downstream of the pre-BCR [2]. Similarly to mature B cell receptors (BCR), signaling from the pre-BCR entails phosphorylation of the ITAMs on tyrosine residues by Src family kinases (SFK), such as Lyn; the phosphotyrosines then serve as docking sites for the dual Src homology 2 (SH2) domains of spleen tyrosine kinase (Syk) [3]. Syk in turn initiates signaling necessary for B cell maturation [2], [4], [5].

The pre-BCR is distinguished from the mature BCR by the fact that the SLC components do not undergo gene rearrangements [2]. Because pre-BCR lack the ability to bind to conventional antigens, one of the major challenges in the field has been to understand their ligand-independent signaling capacity. Although weak basal signals may emanate from Ig*α* and Ig*β* on pre-BCR monomers [2], [6], [7], [8], [9], aggregation is considered essential to generate signals needed for B cell differentiation and survival. Erasmus *et al.* [7] used single particle tracking (SPT) methods to track pre-BCR diffusion and homodimer events on live precursor B cells. These experiments showed that pre-BCR undergo frequent but short-lived dimerization events on the B cell surface. These self-interactions led to autonomous or tonic signaling, including activation of Syk tyrosine kinase and induction of pro-survival B cell lymphoma 6 protein (BCL6), a transcription repressor necessary for pre-BCR to transition into the next developmental stage [10].

Our prior work, reported in Erasmus *et al.* [7], was conducted in the context of precursor B acute lymphoblastic leukemia (B-ALL), which constitutes one of the most common childhood cancers. A significant fraction of B-ALL cases express a functional pre-BCR and exploit the tonic signaling generated from these receptors to survive and proliferate [11], [12]. Inhibitors of Lyn and Syk tyrosine kinases, which are active participants in the pre-BCR pathway, have been shown to negatively affect survival of pre-BCR+ ALL cells, thus highlighting the therapeutic potential of these small molecules in combating B-ALL [7]. Erasmus *et al.* also observed that monovalent anti-VpreB antibody fragments could inhibit pre-BCR dimerization and interrupt receptor-generated survival signals [7].

In addition, that work provided distinct values for receptor diffusion coefficients, as well as homodimer off rates, observed for pre-BCR expressed on the surface of 697 and Nalm6 cells, which were derived from two genetically distinct B-ALL patients [7]. When considered as a whole population (mixture of monomers and oligomers), the pre-BCR on the surface of 697 cells exhibited significantly faster diffusion and had higher dimer off rates as compared to the Nalm6 cells. In the presence of dimer-inhibiting Fab fragments directed at the VpreB, monomer diffusion on both cell lines was similar at 0.16 *μ*m^2^/s (Table 1). These results led us to hypothesize that the difference in dimer off rate could lead to existence of higher order oligomers in Nalm6 cell lines, consistent with the overall slower diffusion rate. However, since receptors are sparsely labeled in SPT experiments, aggregation beyond dimers is difficult to quantify through direct measurements.

Therefore, in order to investigate the existence of these higher order oligomers and the impact of the distinct diffusion coefficients and dimer off rates on signaling events downstream of the pre-BCR receptor, we developed a 3-D, lattice free, spatial, stochastic model of pre-BCR aggregation and tonic signaling. While it aims to describe the dynamics of pre-BCR on precursor B cells, the model presented here integrates elements from previous models of BCR signaling [13], [14], [15] with agent based stochastic simulation methods for spatial simulations [16] as well as rule-based description of signaling networks, in a model framework similar to previous work by our group [17]. The combinatorial model structure includes two extrinsic kinases, the possibility of receptor oligomerization, multiplicity of phosphorylation sites as well as kinase binding and activation mechanisms. This complexity motivated our rule based and “network free” approach. In addition, the transient receptor residency in microdomains leads to receptor “clustering” and that potentially promotes receptor collisions in a sparsely populated landscape. To consider the impact of membrane architecture in the context of live cell SPT data, we implemented the model using a spatially continuous, Brownian motion-based approach [16], [17].

To our knowledge, this is a first attempt to apply the reasonably well understood mechanism of (mature) B cell signaling to tonic signaling in pre-B cells. We relied on our experimental data [7] to the extent possible; however, the simultaneous estimation of the other 20+ parameters in our model pertaining to Lyn and Syk is beyond the capabilities of any one laboratory. For the values listed in Tables 2 and 3, we followed the practice of other modeling groups and relied on parameters estimated from other experimental sources.

The mature BCR signaling pathway has been the focus of much interest, including studies with mathematical models [13], [14], [15]. Features of the pathway include the presence of two different extrinsic kinases (SFK and Syk), both of which have the ability to transphosphorylate, acting on other copies of their own species as well as other substrates such as receptor cytoplasmic tails and signaling components. This provides for several possibly independent positive feedback loops that are sensitive to receptor association either through chemical binding such as dimerization (or oligomerization) or through landscape influenced diffusion resulting in accumulation in membrane domains. Receptor dimerization is often taken into account but is not modelled explicitly [13], [15]. The movement and accumulation of receptors in microdomains is simulated in Mukherjee *et al.* [15]; Tsourkas *et al.* [14] also use a spatial model. In both cases, spatial movement is discretized.

Key parameters in our model, such as diffusion coefficients and dimer off rates, are derived from live cell experiments in Erasmus *et al.* [7]. Apart from coefficients derived from single particle tracking, parameters for the model also include the non-random distribution (i.e., clustering) of receptors. The model simulates receptor diffusion across the cell surface, followed by formation of oligomers and outputs the size and number of receptor aggregates formed explicitly. This contrasts with previous computational efforts to study BCR signaling where receptor aggregation was implicit and the actual size of the oligomers was unknown [13], [15]. Lattice based models of BCR signaling have been used to simulate receptor diffusion in a spatial environment, where the receptors diffuse on a grid like membrane [14], [15]. However, this discretized representation of receptor diffusion on a cell surface complicates the inclusion of smaller molecular species, as well as that of receptor domains, which tend to be dynamic and highly irregular in shape and size. The pre-BCR model presented here is a lattice free model where SPT data was directly used to re-create receptor domains through which receptor diffusion occurs. Finally, the 3-D model enables representation of spatial inhomogeneity existing in the cytoplasm and its effect on cytosolic signaling molecules such as Syk and Lyn.

We report that small differences in receptor dimer off rates affect aggregate sizes and consequent signaling events. Partitioning of the membrane landscape into membrane domains, that transiently trap pre-BCR and promote receptor clustering, influences the frequency of homo-interactions and signaling efficiency. We do not consider alternative scenarios that engage ligands to alter pre-BCR signaling strength, such as self-reactivity to auto-antigens or cross-linking induced when *λ*5 components of the SLC bind to the dimeric stromal ligand galectin-1 [7], [18], [19].

## 2 Methods

We developed a rule-based spatial stochastic model of pre-BCR to investigate tonic signaling occurring downstream of receptor aggregation events. Parameters are summarized in Table 1, 2, 3, and 4, estimated from the literature or from our own experimental measurements [7]. The earliest tyrosine kinases to be engaged in the BCR signal transduction pathway, Lyn and Syk, are explicitly represented in the model. The pre-BCR as well as the two kinases are represented by diffusing particles which may form aggregates and change their phosphorylation state. Below we begin with a brief discussion of our approach in the context of related computational / mathematical models of BCR signaling. Details of the reaction kinetics and rules specifying the interaction of molecules are given, followed by a description of the experimental methods, simulation landscape and molecular movement.

A detailed description of parameter estimation from experimental data as well as a sensitivity analysis presenting the variability of important model outputs to changes in model parameters are given in the Supplement.

### 2.1 Previous Models of BCR Signaling

In this work, we simulate the spatial movement of individual receptor, Lyn, and Syk molecules in a spatially continuous model. We simulate the binding and phosphorylation states of the two Ig*α*, Ig*β* chains on each receptor, as well as those of the Lyn and Syk molecules. We are not aware of prior mathematical models aimed at tonic signaling from pre-BCR. We relied on several recent models of (mature) BCR signaling to develop our model, in particular Barua *et al.* [13], Tsourkas *et al.* [14], Mukherjee *et al.* [15]. These works focus on different aspects of BCR signaling, but they all share some of the core elements. They include the receptor, Syk, at least one Src family kinase (typically Lyn), and account for their various activation/phosphorylation states. The action of the various kinases *associated with the same receptor* are described similarly.

The models differ to some extent in terms of including additional elements: Mukherjee *et al.* [15] includes a phosphatase; Barua *et al.* [13] includes an additional Src family kinase, and two additional downstream species; Tsourkas *et al.* [14] has a detailed model of receptor-antigen binding.

There are important differences when it comes to spatial resolution, receptor oligomerizations, and the implementation of trans-phosphorylation (when a kinase associated with one receptor acts upon a substrate on a different receptor). Barua *et al.* [13] employs a non-spatial deterministic (ODE based) model that is defined in a rule-based approach. The model has a very large number of chemical species (1122) and reactions (22388) due to the three additional species they include. Receptor oligomerization is not modeled explicitly. Trans-activation/phosphorylation is modeled in terms of second order reactions.

The models in Tsourkas *et al.* [14] and Mukherjee *et al.* [15] are stochastic and particle based in that they simulate the evolution of the number of copies and movement of each chemical species through individual reaction / diffusion events. Mukherjee *et al.* [15] use a spatially coarse-grained model where the simulation area is divided into compartments that are considered well-mixed; second order reactions may occur within compartments. They separately simulate the formation of spatial concentrations of receptors (referred to as clusters) and signaling scenarios on readily formed clusters. They also allow for receptor dimers. Some trans-phosphorylation interactions are modeled as second order reactions. Tsourkas *et al.* [14] explore the capacity of the BCR-Lyn-Syk pathway to discriminate antigen binding strength. They use receptor dimerization as a requirement for receptor phosphorylation but do not explicitly represent dimers. They explicitly represent antigen, receptor, Lyn and Syk as base species in a simplified model of BCR signaling. The movement of individual molecules is represented on a rectangular grid (lattice).

All of the models discussed above have a level of detail appropriate for their specific purpose. Our goal is to integrate recent insights from single particle tracking of pre-B cell receptors [7] with elements of BCR signaling that are present in pre-B cells, into a realistic model of tonic (antigen independent) pre-BCR signaling. We have direct experimental information on the dynamics of receptor dimerization, as well as on features of the membrane landscape that favor the accumulation (concentration) of receptors, and this motivated the choice of a fully detailed (continuous) spatial movement and explicit inclusion of receptor aggregation.

Since our framework represents the topology (geometric structure) of receptor oligomers and associated Lyn and Syk molecules, we represented trans-phosphorylation as first order reactions made possible by the structure of these molecular aggregates. Allowing aggregation of receptors results in a fairly large number of species. This requires an agent- and rule-based approach where the simulation keeps track of the location and binding state of each copy of the three base species (receptor, Lyn, Syk). Below we describe our model in detail.

### 2.2 Rules Representing pre-BCR Signal Initiation

Although the pre-BCR and BCR are expressed at distinct time points in the lymphocyte development and contain structurally different light chains, both these receptors enlist the same set of Src and Syk family kinases to initiate signaling [20], [21]. The pre-BCR propagates signaling through the non-covalently attached Ig*α* and Ig*β* units which each contain ITAMs [2]. The ITAMs contain tyrosine residues (Y182 and Y193 on Ig*α* and Y195 and Y206 on Ig*β*) that are substrates for phosphorylation by the kinases [22], [23].

In the model, the divalent pre-BCR receptors form dimers that may subsequently engage with additional pre-BCR to form higher order, linear oligomers through their free receptor binding domains (Table 1). Receptor aggregation is triggered through molecule collision events and receptor unbinding is intrinsically triggered with a probability consistent with the experimentally measured dimer off-rate and the time step.

Ligand independent receptor aggregation in pre-BCR is followed by binding of Lyn to the ITAMs [2]. Lyn has four structurally distinct domains through which it interacts with the ITAMs [24], [25]. The unique domain of Lyn is known to constitutively associate with receptors and bind to non-phosphorylated Ig*α*, whereas the SH2 domain binds to phosphorylated ITAMs on both Ig*α* and Ig*β* [26], [27]. Although Lyn has been reported to bind ITAMs in both signaling subunits, it preferentially binds to Ig*α* at least twice more likely than to Ig*β* [28].

In the model, Lyn can bind to a free ITAM site on a receptor (Table 2). The receptor may be part of an aggregate or a single receptor by itself. Lyn can bind to an unphosphorylated Ig*α* through its unique domain and bind to a phosphorylated Ig*α* or phosphorylated Ig*β* through its SH2 domain. Only one Lyn molecule is allowed to bind per Ig*α* or Ig*β*. Similarly, to receptor-receptor binding, Lyn-receptor binding events are collision triggered while unbinding occurs with a probability calculated through off rates and the time step. The two phosphorylation sites on Ig*α* are represented as a single site with phosphorylation status- 0, 1 or 2. The phosphorylation sites on Ig*β* are treated similarly.

Lyn association with the receptors may be followed by receptor phosphorylation: the presence of at least one Lyn bound to any receptor in an aggregate is sufficient for initiating phosphorylation of other receptors in the same aggregate [33]. Lyn itself can also become transphosphorylated (Y397) by other Lyn molecules, which results in an increase of Lyn kinase activity [33], [34], [35], [36].

In the model, Lyn docked on a receptor can phosphorylate Lyn on a nearby receptor, i.e., the two receptors must be immediate neighbors in the same receptor complex (Table 3). Phosphorylation rates depend on the phosphorylation state of Lyn. Similar to Lyn-Lyn phosphorylation, Lyn must be on an immediately adjacent receptor in the same complex in order to phosphorylate the ITAMs. Phosphorylated Lyns are assumed to be activated and have ~3 times stronger kinase activity. The Lyn phosphorylation site can have the phosphorylation status- 0 or 1.

The phosphorylated ITAMs form docking sites for the Syk [32]. While Syk can bind to both mono-phosphorylated and doubly-phosphorylated ITAMs through its SH2 domains, the binding affinity is significantly higher for doubly phosphorylated ITAMS; this is reflected by high dimer on-rate and lower off-rate [31]. Binding of Syk to Ig*α* is three times more than the binding seen to Ig*β* [32].

In the model, free Syk can bind to any one of the two ITAMs within the Ig*α* and Ig*β* heterodimer of each pre-BCR complex upon collision. Each ITAM in the heterodimer is regarded as a Syk binding site and each can bind Syk independently of each other. However, since Lyn binding is also possible on the same sites, Syk molecules cannot bind the ITAMs if they are occupied and the same rule is applied for Lyn molecules. Syk dissociation from the receptor also occurs through an intrinsically triggered unbinding probability which is determined through the Syk off rate and the time step. The Syk off-rate is higher for doubly phosphorylated ITAMS versus singly phosphorylated.

Syk can undergo phosphorylation on specific tyrosine residues in its catalytic domain (Y519 and Y520) by another Syk docked on adjacent ITAMS in the same BCR or pre-BCR aggregate [37], [38]. Syk can also undergo phosphorylation by a Lyn docked on adjacent ITAMs in the same BCR or pre-BCR aggregate molecule in its linker regions (Y342 and Y346) [37].

In the model, Syk or Lyn molecules must be on an immediately adjacent receptor in the same complex in order to phosphorylate other Syk molecules (Table 3). Syk molecules can phosphorylate other Syk molecules in their catalytic domain and Lyn molecules can phosphorylate other Syk molecules in their linker regions. Syk molecules that have been phosphorylated in their catalytic domain are assumed to have stronger kinase activity. The two phosphorylation sites on the catalytic domain have been lumped together and can have the phosphorylation status- 0 or 1; the two phosphorylation sites on the linker region are treated similarly.

Receptor, Lyn and Syk dephosphorylation is based upon probabilities that takes into account the molecule’s dephosphorylation rate and the time step (Table 4).

### 2.3 Experimental Methods

As described in Erasmus *et al.* [7] receptor diffusion coefficients for 697 and Nalm6 cells were derived from measurement of receptor trajectories using SPT imaging technique which involves the labeling and tracking of particles using a microscope [7], [39]. For particle labeling during SPT measurements, monovalent anti-Ig*β* Fabs’ against the Ig*β* subunit of the Pre-BCRs were generated using hybridomas [7]. These anti-Ig*β* Fabs’ were labeled with streptavidin-conjugated multicolor quantum dots (QD), which enable the fluorescent tagging and tracking of molecules across membrane surfaces at nanometer resolutions [7], [40]. The two QDs used for imaging were QD585 and QD655. The monovalent QD probes were then allowed to bind pre-BCR and their movement was tracked on a live membrane using SPT imaging [7]. The two color QD labeling enabled the identification of pre-BCR dimers on the membrane surface.

### 2.4 Simulation Space and Membrane Landscape

**Fig. 1.**
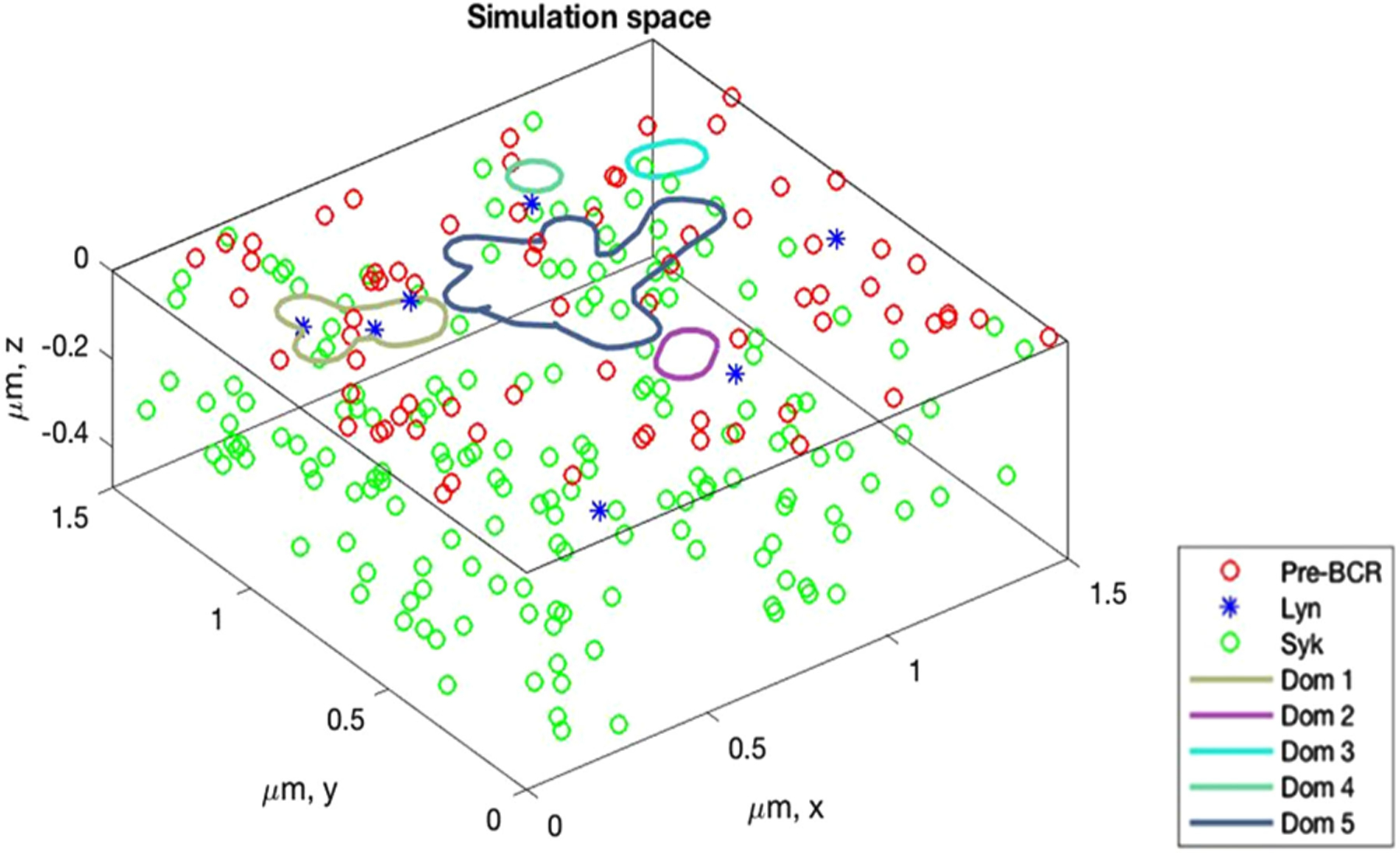
3-D simulation space containing pre-BCR (red circles), pre-BCR specific domains (enclosed lines), available Lyn (blue asterisks) and Syk molecules (green circles). Pre-BCR and Lyn molecules undergo Brownian motion in the x and y planes and these planes represent the plasma membrane. Syk molecules undergo Brownian motion in the x, y and z planes and these planes represent the cytoplasm and the plasma membrane. Pre-BCR molecules are free to enter their domains in the plasma membrane but pay an exit penalty to leave the domains. Lyn and Syk molecules freely diffuse and do not observe any domains. There are 71 pre-BCR molecules in the system, 7 Lyn molecules (total number of Lyn molecules available to the receptor for binding) and 169 Syk molecules (697 cells) or 959 Syk molecules (Nalm6 cells).

We created a three-dimensional (3-D) simulation space to represent interactions between the pre-BCR and Lyn, which are both plasma membrane residents, and Syk in the cytosol. Thus, the two-dimensional (2-D) membrane is populated with transmembrane pre-BCR and inner leaflet-bound Lyn molecules, while the 3-D cytosol is populated with Syk molecules (Fig. 1). The membrane contains pre-BCR specific domains and receptors can diffuse across domains and domain-free areas. The domains were identified through analysis of two-color SPT. In order to extract the receptor domain sizes and contours from SPT, data sets containing the particle trajectories were subjected to the domain reconstruction algorithm (DRA), which was previously developed and used in Pryor *et al.* [17] Briefly, the DRA reads in the SPT trajectories and ranks their points into slow moving (confined) or fast moving points (free) using their jump sizes over different time intervals. The confined points are postulated to be in a domain (such as lipid rafts, protein domains or corrals) that impede the movement of the particles. We then cluster the slow-moving points into groups based on whether their distance from each other is less than the reference distance, *L*. After cluster identification, we build contours around them that represent the boundaries of receptor domains.

From morphometric measurements, pre-B cells were estimated to have a total cell surface area of 315.7 *μ*m^2^ and a cytosolic volume of 321.8*μ*m^3^. The pre-B cell radius is ≈ 5 *μ*m and nuclear radius is 3.7*μ*m. In our simulations, the pre-B cell membrane landscape is represented by a rectangular cuboid with an area of 2.25 *μ*m^2^ (1.5 *μ*m × 1.5 *μ*m) and volume of 1.25 *μ*m^3^ (depth of 0.5 *μ*m). The total number of receptors is equivalent in both the cell lines (10000 receptors) for a density of ≈ 32 receptors/*μ*m^2^. For our simulation area, this resulted in 71 receptors occupying the membrane landscape.

The amount of Syk varied between the cell lines. Based upon calibrated western blotting experiments, we estimate that 697 cells have ~ 48000 Syk molecules while Nalm6 cells express ~274000 Syk proteins (Table 1). For our simulation volume, this resulted in 169 Syk molecules for 697 cells and 959 Syk molecules for Nalm6 cells. The amount of Lyn available to the receptors is estimated to vary between 5% and 10% of the total receptors present in the cell [34], [41]. Faeder *et al.*, in their investigation of signals emanating from Fc*ε*RI, modeled the pool of Lyn available to the receptors as 7% of the total receptor concentration and assumed that all of the available Lyn is in a form, which when bound to the receptors, is capable of initiating phosphorylation events [29]. We apply the same principles in our model, where we assume that the total amount of Lyn available to the receptors would be at most 10%. This would mean that at any given time in the pre-B cell, only 1000 Lyn molecules are available for interaction with the 10000 pre-BCRs. For our simulation area, this amounts to 7 Lyn molecules available for interaction with a total number of 71 receptors.

### 2.5 Molecule Diffusion

In our simulation, molecules undergo Brownian motion in the x, y and z plane. Lyn and pre-BCR undergo diffusion in the x and y plane only, whereas Syk molecules can also diffuse in the z plane representing the cytosol.

Receptor jumps $\text{Δ}\vec{r}=\vec{r}(t+\text{Δ}t)-\vec{r}(t)$ are generated by choosing a random number from a normal distribution and processing it with the root mean square (RMS) displacement of the molecule to generate the new coordinates with the time increment of Δt The RMS displacement is given by: $\text{RMS}=\sqrt{\text{Δ}r^{2}}=\sqrt{2D_{\text{agg}}\text{Δ}t}$ The new coordinates are computed as follows [16], [17], [42]:

$$\begin{array}{l} \vec{r}(t+\text{Δ}t)=\vec{r}(t)+\text{RMS}\cdot \left(\xi_{x},\xi_{y}\xi_{z}\right)\\ \;x(t+\text{Δ}t)=x(t)+\text{RMS}\cdot \xi_{x}\\ \;\to \;y(t+\text{Δ}t)=y(t)+\text{RMS}\cdot \xi_{y},\\ \;z(t+\text{Δ}t)=z(t)+RMS\cdot \xi_{z} \end{array}$$

where *x*, *y*, *z* represent the molecule’s Cartesian coordinates and *ξ*_*x*_, *ξ*_*y*_, *ξ*_*z*_ are random numbers chosen independently from a standard normal distribution: $f(\xi )\propto \exp\left(-\xi^{2}/2\right)$; ξ=0, $\xi^{2}=1$.

Since pre-BCRs can form dimers as well as higher order oligomers, in the model we assume that the diffusion coefficient *D*_agg_ of a pre-BCR complex is inversely proportional to the size of the complex: $D_{\text{agg}}=D_{\text{rec}}/M_{\text{agg}}$. The size *M*_agg_ of the complex reflects the number of receptors in an aggregate and *D*_rec_ is the diffusion coefficient of a single, unbound receptor.

### 2.6 Boundary Conditions and Probability of Escape

For any finite simulation space, boundary conditions need to be specified so that particles remain in the simulation area or volume. For the pre-BCR, we apply periodic boundary conditions at the edges of the simulation space [17]. When a receptor reaches the edge of the simulation space (in the *xy* plane) and the receptor jump calculated displaces the receptor outside the simulation space, we divide the jump into two segments. The first segment displaces the receptor to the edge and the second segment is calculated from the opposite edge of the simulation space, such that the receptor re-enters the simulation space from the opposite boundary. For receptors transiently trapped in domains, we apply reflective boundary conditions [17]. As in Pryor *et al.*, the receptor jump is divided into two segments: one segment displaces the receptor to the edge of the domain and the second displaces it outside of the domain [17]. When the receptor reaches the edge of the domain, a probability for escape from the domain is calculated and if the probability of escape is not met, then the receptor is reflected back into the domain with the remaining segment. If the probability of escape is met, then the receptor continues out of the domain. An exit penalty limits receptor escape from the domains; this penalty was obtained from SPT experimental data by calculating the ratio of the membrane area explored by the slow-moving single particles versus the membrane area explored by fast moving single particles. The exit probability for receptors in the pre-B cells was found to be 0.2. We apply the same periodic boundary conditions to Lyn and Syk molecules in the *xy* plane. For Syk, we also apply reflective boundary condition in the *z* direction, such that when the receptor reaches edge of the simulation space, it is reflected back into the cytosol.

### 2.7 Reaction Kinetics

For molecular reactions, we chose reaction kinetics similar to those used in the spatial stochastic simulator Smoldyn, which uses Smoluchowski dynamics with revisions to implement reaction events [16]. In essence, there are two different ways to simulate reaction events depending on whether they are first or second order reactions. First order reactions include molecule phosphorylation, dephosphorylation and molecule dissociation. If the reaction rate is *k*_first–order_ the probability of any of these first order reaction events occurring over a time step Δ*t* is given by [16], [17]:

$$P_{\text{first-order}}=1-\exp\left(-k_{\text{first}-\text{order}}\cdot \text{Δ}t\right)\approx k_{\text{first}-\text{order}}\cdot \text{Δ}t$$

The approximate value is appropriate for small time steps (in our simulations we used $\text{Δ}t=10^{-5}\text{s}$).

Second order reactions (of the form A+B→…) include molecule association events such as receptor dimerization or aggregation into higher order oligomers and receptor binding of molecules such as Lyn and Syk. For these reactions, a parameter called binding radius *σ*_*B*_ is used to determine the outcome of a collision event. A binding event will take place if and only if two eligible molecules are within *σ*_*B*_ of each other. The binding radius for kinases was calculated using Smoldyn, taking into account the on-rate of the reaction, diffusion coefficients and the time step. The binding radius for a pair of receptors was determined using SPT data from Erasmus *et al.* [7] as initial parameters for well-mixed Matlab simulations and spatial stochastic simulations with no domains (Supplement 1). Briefly, we ran well-mixed Matlab simulations with an estimated on-rate and estimated dimer off-rate for pre-BCR aggregation in 697 cells. This produced a ratio of monomers, dimers and higher order oligomers. To set the binding radius, we then used the estimated dimer off-rate with varying binding radii in a domain-free spatial stochastic simulation (coded in Fortran) to obtain the same ratio of monomers, dimers and higher order oligomers as observed in the well-mixed Matlab simulations (Table 1). We varied the binding radii between 3.5 × 10^−4^ *μ*m to 5 × 10^−5^ *μ*m in the spatial simulations to match the well-mixed predictions. The closest binding radius that matched was 1.0 × 10^−4^ *μ*m (Supplementary Fig. 2, which can be found on the Computer Society Digital Library at http://doi.ieeecomputersociety.org/10.1109/TCBB.2022.3166149) and this estimated binding radius was used as the dimer on rate for the two cell lines.

### 2.8 Simulation Runs and Processing

**Fig. 2.**
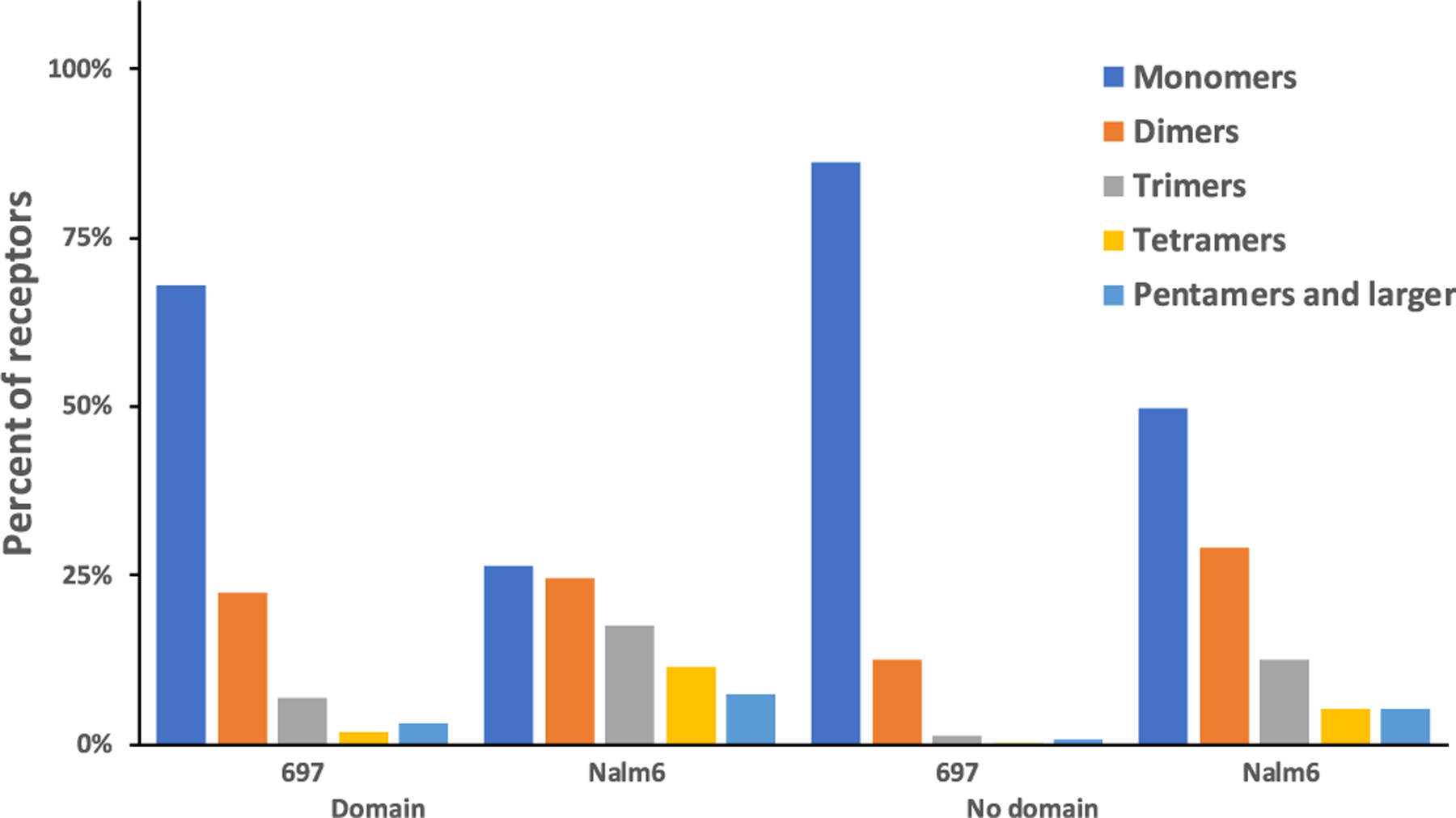
Percent of receptors engaged as monomers or different-sized oligomers in 697 and Nalm6 cells (with and without domains) at steady state. All bars are the averages of 3 runs between 50 and 600 seconds. The binding radius for the two cell lines is 0.0001 *μ*m. The dimer off-rate for 697 cells is 1.14/s, compared to 0.159/s for Nalm6 cells‥

We performed 3 simulation runs for each of the 4 conditions discussed below. The values reported in Figs. 2, 4, 6, and 8 are derived from time-based averages extracted from individual simulations.

**Fig. 3.**
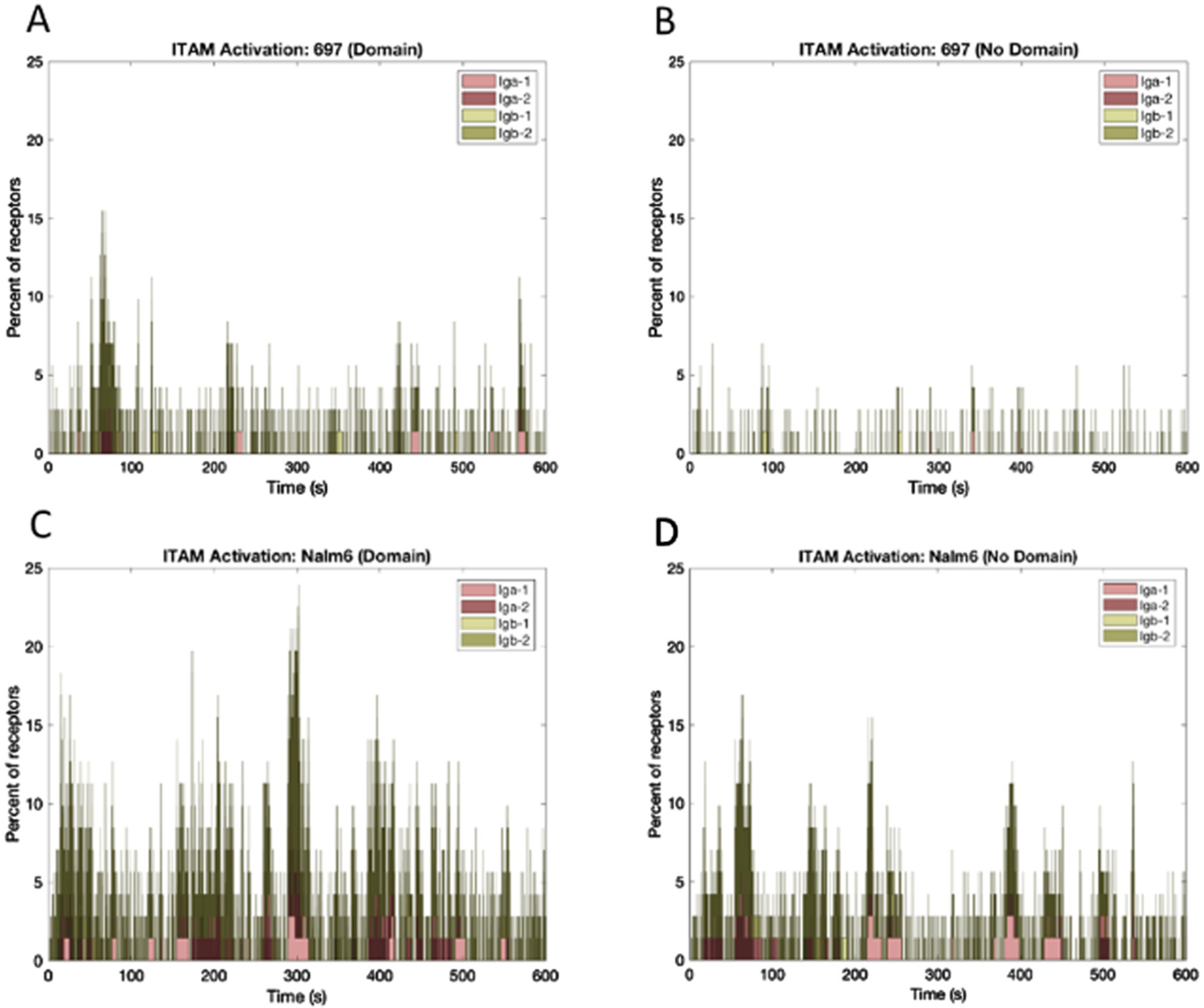
Time evolution of the percent of Ig*α* and Ig*β* phosphorylation on the pre-BCR for 697 and Nalm6 cells (with and without domains). (A) 697, Domain. (B) 697, No Domain. (C) Nalm6, Domain. (D) Nalm6, No Domain. In the model, the two phosphorylation sites on each ITAM Ig*α* and Ig*β*) are lumped together such that Ig*α* can be phosphorylated singly, doubly or unphosphorylated. Ig*β* can also be phosphorylated singly, doubly or unphosphorylated.

For each simulation run, the initial positions for each particle are drawn independently from uniform distributions within the simulation space. Typical simulations are run for a total simulation time of *T* = 600 *s*, of which the first *T*_*ini*_ = 50 *s* are set aside for equilibration. The averages reported were based on the remaining simulation time (typically ~ 450 seconds per run). Each of the average quantities of interest is derived from the time series of the number of molecules / instances (such as receptor-bound Lyn, etc.) as illustrated in Figs. 3, 5, and 7. Corresponding standard deviations were computed for each run, and also from the averages of individual replicates.

## 3 Results

### 3.1 Impact of Varying Dimer Off Rate and Domains on Receptor Aggregation

From SPT measurements, Erasmus *et al*., had observed that pre-BCR on 697 cells exhibited a higher dimer off-rate as compared to Nalm6 cells. This led to the speculation that pre-BCR on the surface of Nalm6 cells were forming higher sized oligomers, supported by observed cases of SPT that captured apparent “dimers” of pre-BCR tagged quantum dots that exceeded the theoretical distance of 100 nm for a minimal pre-BCR dimer. We populated our spatial stochastic model with the estimated off-rates in order to observe whether the two cell lines formed different sized aggregates. Fig. 2 compares the predicted aggregate sizes formed on these two distinct leukemia cell lines. Model predictions support the hypothesis that pre-BCR on Nalm6 cells forms much higher order oligomers when compared to receptors on 697 cells. At steady state, the majority of pre-BCR are in the monomer state on 697 cells, compared to only 25% monomers predicted for pre-BCR in a domain-studded membrane landscape. Thus, the spatial stochastic model provides evidence that the rate of higher order oligomer formation may be linked to variations across individuals that influence the off-rate or membrane landscape, such as the single nucleotide polymorphisms implicated in our earlier work [7].

Pre-BCR expression is relatively low, at approximately 10000 receptors per cell [7]. However, the typical clustering of pre-BCR in small nanoscale domains can create locally high densities that may be important to aggregation kinetics. As outlined in Methods, we used data gathered from SPT measurements to re-create pre-BCR domains in our *in-silico* membrane landscape. Fig. 2 shows results predicting impact of domains on the percent of receptors engaged in aggregates of different sizes for 697 and Nalm6 cells. Although we found that while the presence of domains increased the size of pre-BCR aggregates in both cell lines, 697 cells are predicted to have a greater dependency on the domains to promote homo-interactions (Fig. 2).

### 3.2 Impact of Varying Dimer Off Rate and Domains on Receptor Activation

BCR and pre-BCR aggregation leads to phosphorylation of tyrosine residues on the ITAMs in Ig*α*/Ig*β* accessory chains by Lyn, as well as Lyn-Lyn transphosphorylation [33], [36]. In our model, Lyn can phosphorylate other receptors within the same aggregate; Lyn can also be trans-phosphorylated by other Lyn molecules, provided they all reside on the same aggregate. We simulate this relationship by providing a receptor-bound Lyn access to receptors for phosphorylation that are one bond adjacent on each side within an aggregate. Since the sizes of the oligomers formed in the two different cell lines differed, we wanted to investigate the effect of aggregate sizes on ITAM phosphorylation. Fig. 3A-D display the simulated levels of Ig*α* and Ig*β* phosphorylation, which vary over time in these stochastic simulations. Results indicate that the presence of domains enhances the levels of receptor phosphorylation in both cell lines, with Nalm6 reaching a higher baseline value and exhibiting higher peaks consistent with the longer interaction lifetime.

**Fig. 4.**
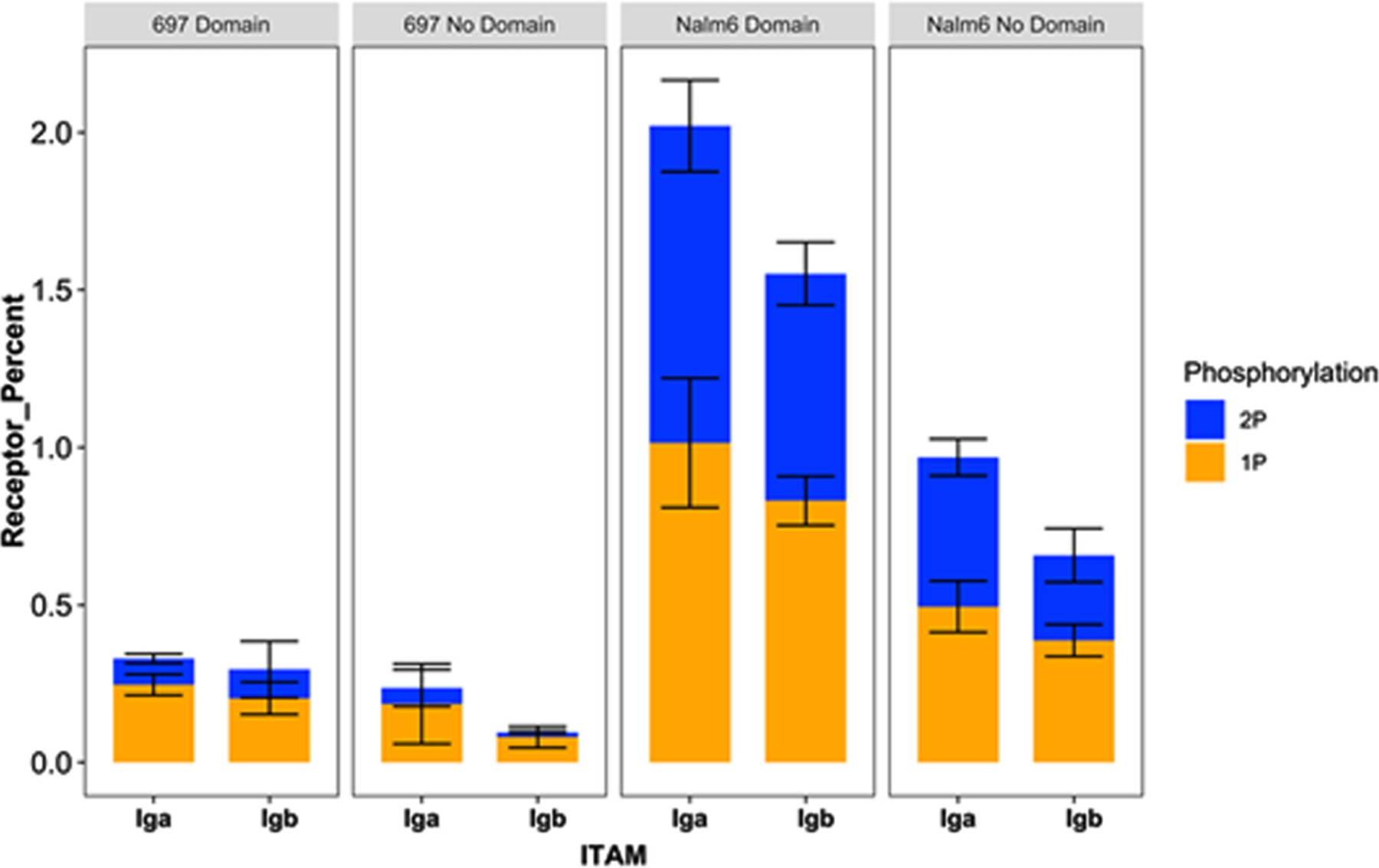
Percent of average Ig*α* and Ig*β* phosphorylation status for pre-BCR (697 and Nalm6 cell lines, with and without domains) at steady state (average of 3 runs between 50 and 600 seconds, ± standard deviation). Bars reporting singly and doubly phosphorylated ITAM bars for Ig*α* and Ig*β* are stacked.

Fig. 4 presents a summary comparison of the average phosphorylation state for Ig*α* and Ig*β* across the two cell lines. The bar graphs indicate that only 0.20–0.25% of Ig*α* / Ig*β* ITAMs are likely to achieve the mono-phosphorylation state during tonic signaling in 697 cells, with an even lower percent (<0.1%) achieving the double-phosphorylation state. In contrast, up to 1% of Ig*α*/Ig*β* ITAMs are predicted to be singly phosphorylated at steady state in Nalm6 cells.

### 3.3 Impact of Varying Dimer Off Rate and Domains on Lyn Binding and Phosphorylation

**Fig. 5.**
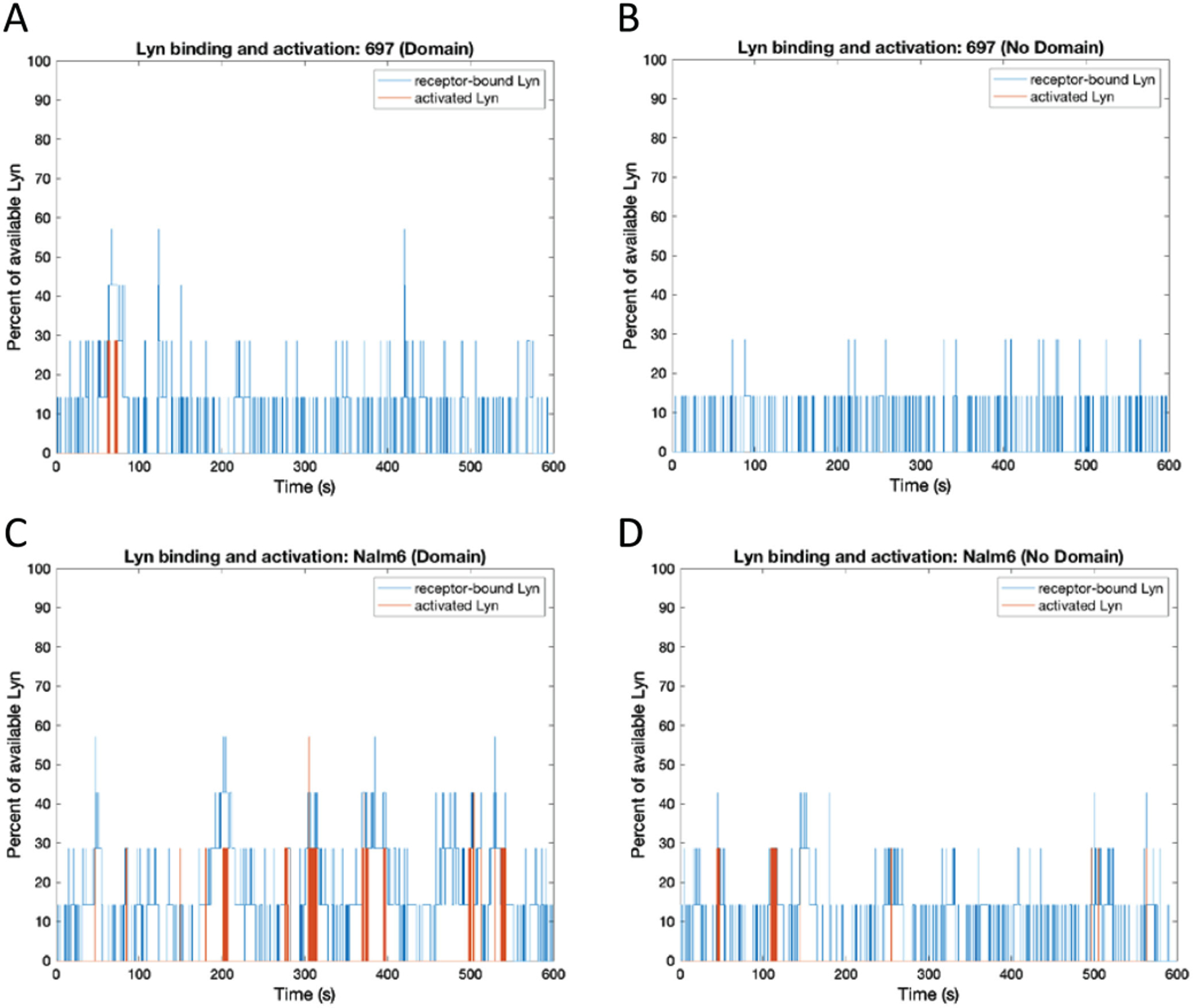
Time evolution of the percent of total available Lyn bound to the receptor and activated Lyn for the 697 and Nalm6 cell line (with and without domains). (A) 697, Domain. (B) 697, No Domain. (C) Nalm6, Domain. (D) Nalm6, No Domain. The total amount of Lyn molecules available to the receptor for binding is 7 Lyn molecules for both cell lines. The total simulation time for each run was 600 seconds. Activated Lyn represents a Lyn molecule which has been phosphorylated at Y397 by another Lyn molecule.

**Fig. 6.**
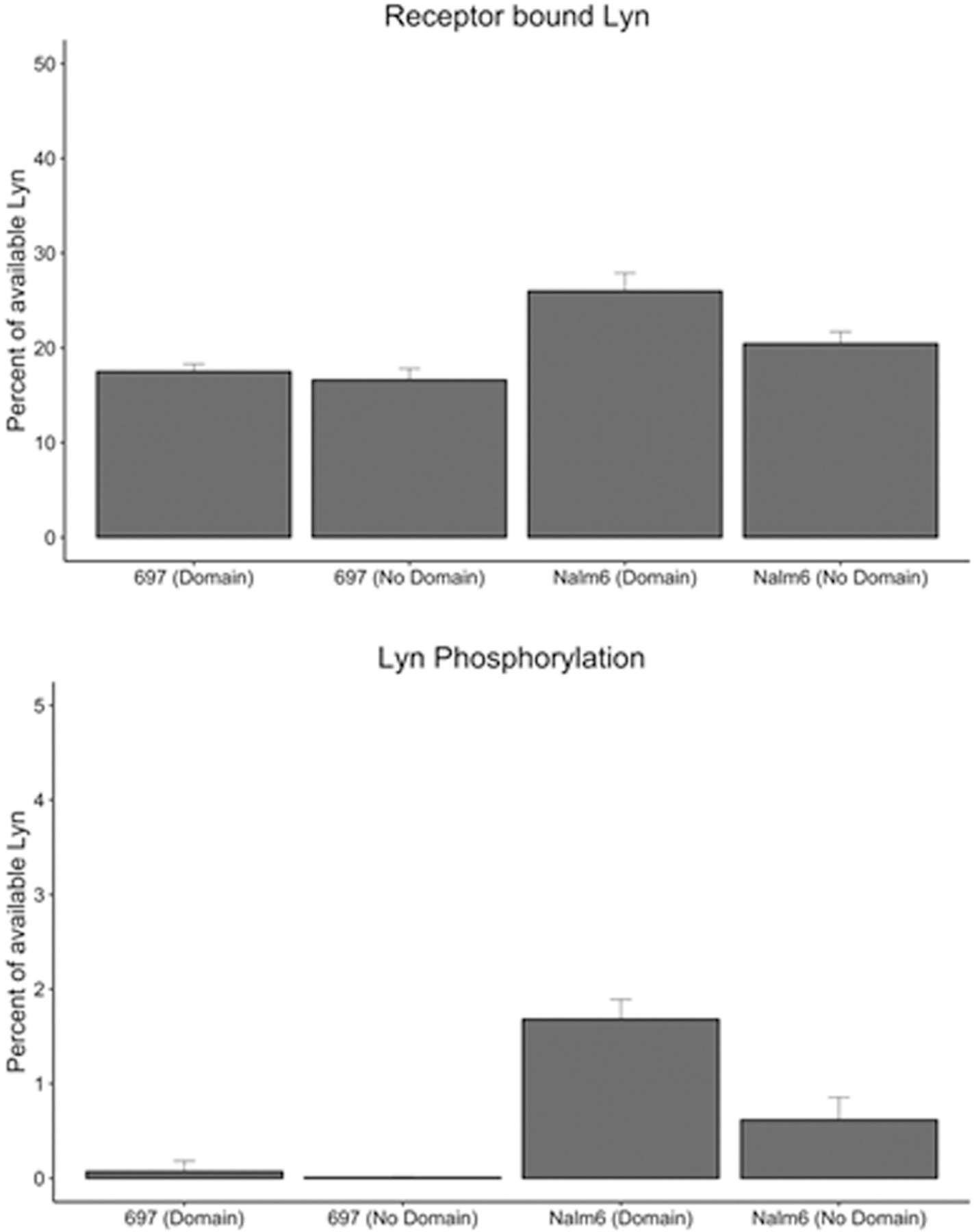
Percent of total available Lyn bound to the receptor and activated Lyn for the 697 and Nalm6 cell line (with and without domains) at steady state (average of 3 runs between 50 and 600 seconds, ± standard deviation). (A) Percent of receptor bound Lyn. (B) Percent of Lyn phosphorylated. The amount of Lyn available to receptors is 10% of the total receptor population.

We investigated whether the different dimer off-rates between the cell lines and the presence of domains have an effect on the amount of Lyn recruited to the receptors, as well as their phosphorylation/activation status. Our simulation conditions provide no penalty for Lyn to enter or leave pre-BCR domains. Under these conditions, Figs. 5 and 6 predict that the average amount of Lyn molecules recruited to the receptors is comparable under all simulation conditions in the same cell background, regardless of domains. However, Fig. 6 indicates that the amount of Lyn activation is strongly linked to the higher oligomeric state of the pre-BCR in Nalm6 cells, which is promoted by domains (Fig. 2).

### 3.4 Impact of Varying Dimer Off Rate and Domains on Syk Binding

**Fig. 7.**
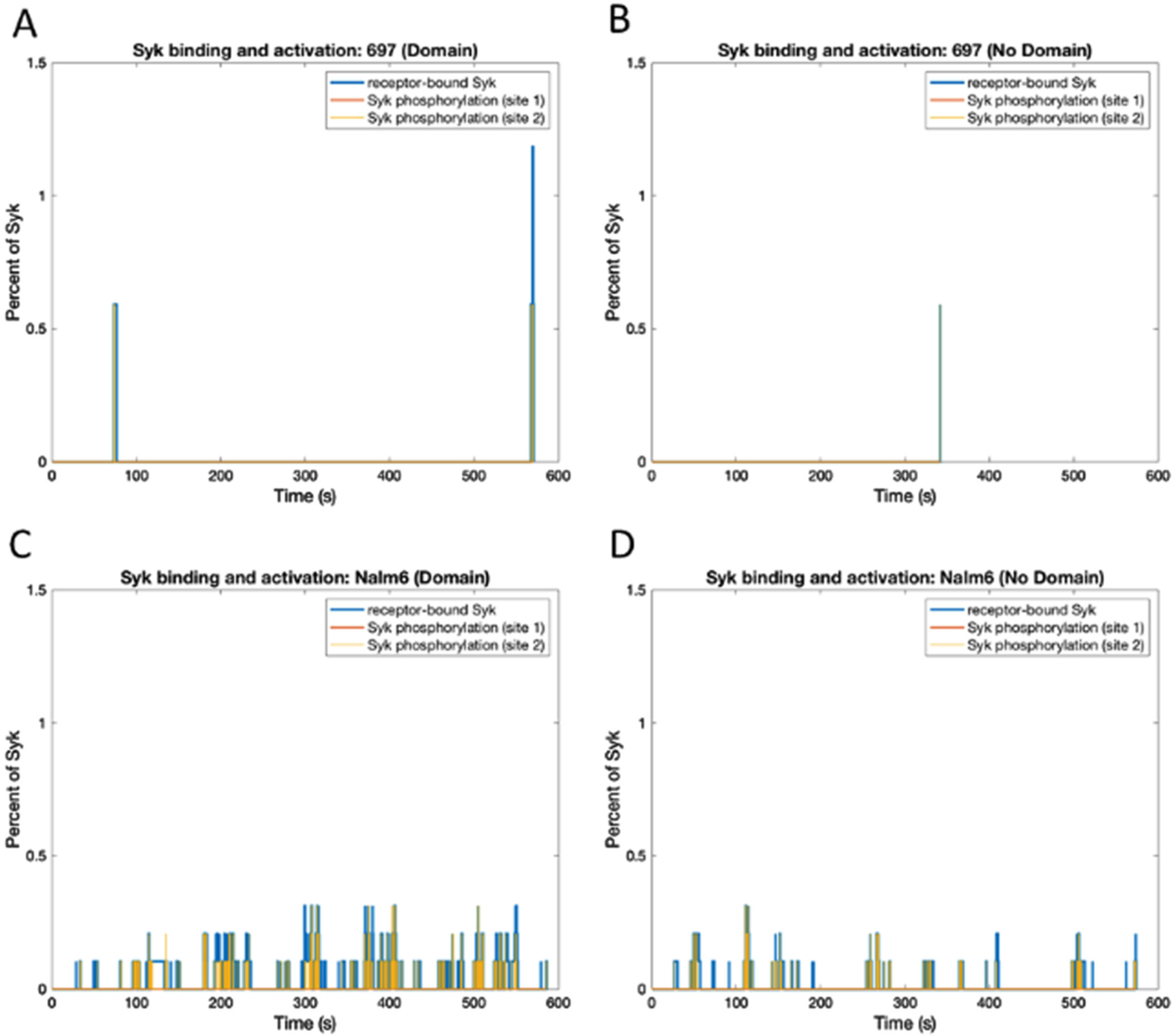
Time evolution of the percent of total Syk molecules bound to the receptor and activated Syk for the 697 and Nalm6 cell line (with and without domains). (A) 697, Domain. (B) 697, No Domain. (C) Nalm6, Domain. (D) Nalm6, No Domain. Syk phosphorylation site 1 represents Syk transphosphorylation in its catalytic domain (Y519 and Y520) by another Syk molecule. Syk phosphorylation site 2 represents Syk phosphorylated in its linker region (Y342 and Y346) by Lyn. The two phosphorylation sites on the catalytic domain of Syk (site 1) have been lumped together and can be phosphorylated or unphosphorylated, as have the two phosphorylation sites on the linker region of Syk (site 2)‥

**Fig. 8.**
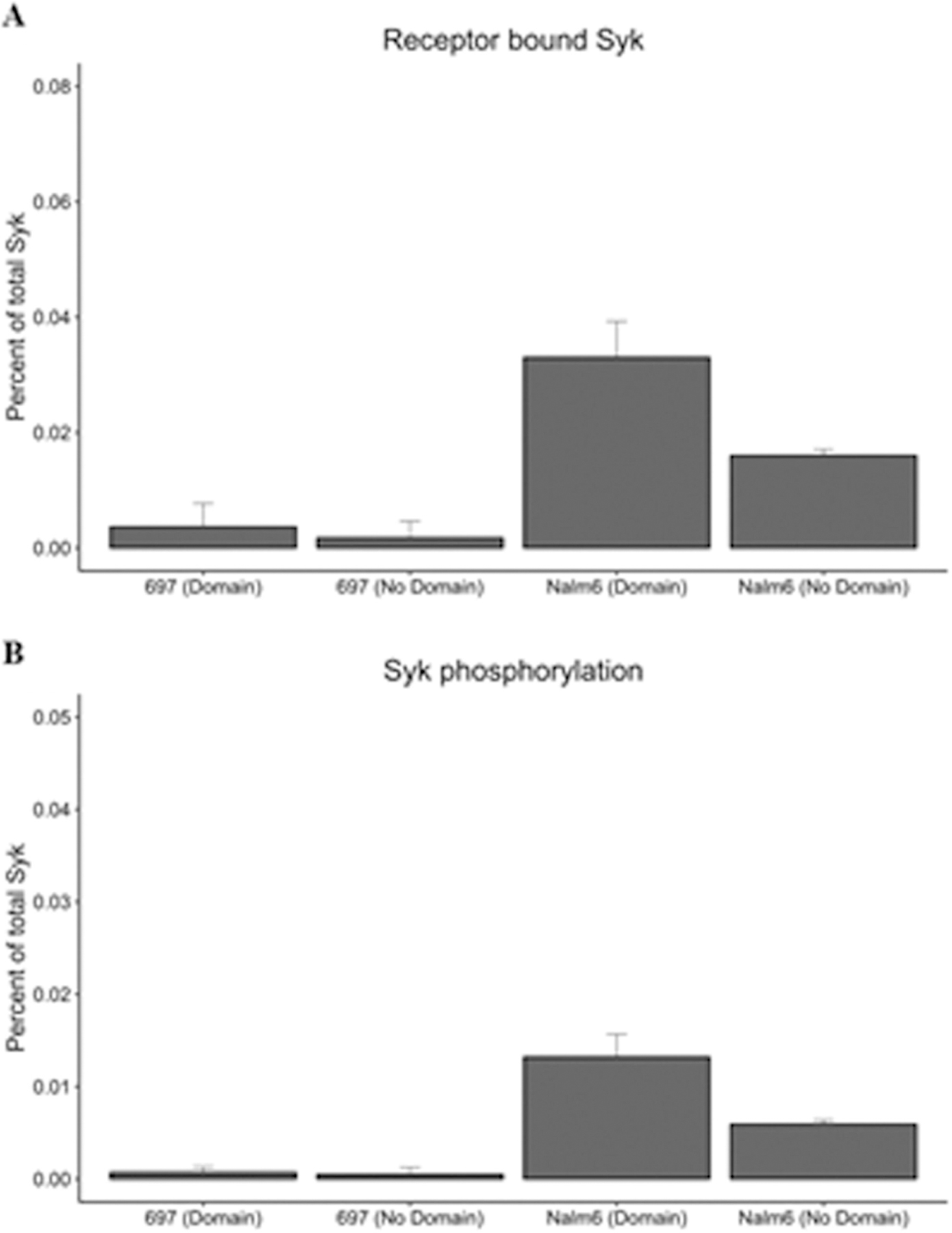
Percent of total available Syk molecules bound to the receptor and activated Syk for the 697 and Nalm6 cell line (with and without domains) at steady state. Syk binding and phosphorylation at steady state. (A) Percent of receptor bound Syk. (B) Percent of Syk phosphorylated by Lyn. Bars report averages of 3 runs (3 runs between 50 and 600 seconds, ± standard deviation).

Signal propagation following ITAM phosphorylation is dependent on recruitment of Syk [32]. We next investigated the effects of different dimer off-rates and receptor domain residency on Syk associations with receptors, as well as on Syk phosphorylation status. Other parameter variations include differences in expression levels in the two different cell lines, where Nalm6 cells have more than five times the number of Syk molecules compared to 697 cells. Simulation results for Syk binding and phosphorylation under these varying conditions are reported in Figs. 7 and 8. Notably, Syk recruitment and phosphorylation are predicted to be rare events in 697 cells (Figs. 7A and 7B). Stochastic simulations of Syk binding and activation in Nalm6 cells, which have the advantage of longer dimer lifetimes and greater Syk abundance, predict that Syk molecules engage more frequently with receptors and further enhanced by membrane domains (Figs. 7C and 7D). Up to 0.033% of Syk are likely to be engaged with receptors at steady state in Nalm6 cells. At ~274000 Syk molecules per cell, this translates to recruitment of approximately 90 Syk molecules. Less than half of these are predicted to be phosphorylated at steady state.

### 3.5 Statistical Considerations

The main results reported are averages of direct outputs of simulations. The core set of simulations were performed for 4 conditions (two different cell lines, with / without domains), with 3 replicates for each condition. Each run used a total simulation time of 600 seconds; the first 50 seconds were set aside to ensure statistical equilibrium.

We computed standard deviations corresponding to equilibrium fluctuations within each simulation as well as variations between the averages of the three replicates. The equilibrium fluctuations reflect relatively broad distributions that are characteristic of a dynamical stochastic equilibrium. For all the observables (receptor aggregate sizes, fraction of bound Lyn / Syk, fraction phosphorylated), the equilibrium fluctuations were significantly larger than the variability between replicates. We report the latter in Figs. 4, 6, and 8.

A comparison of the mean values and the replicate based standard deviations indicates that our conclusions about the impact of cell type (as reflected by the receptor off rate) and presence of domains on receptor aggregation and phosphorylation is statistically significant. The effect of the cell type on Lyn and Syk binding, and phosphorylation is also significant. The impact of domains on Lyn and Syk binding and phosphorylation is significant for the Nalm cell line, but less so for the 697 cell line.

## 4 Discussion

In this study, we developed a spatial stochastic model to explore autonomous signaling between pre-BCR on the surfaces of two different leukemia cell lines (697 and Nalm6). Our results link marked differences in aggregate sizes and the phosphorylation levels of receptors and their signaling partners to the experimentally measured differences in pre-BCR homo-interaction off-rates. We show that transient co-confinement of pre-BCR within plasma membrane microdomains impacts the pre-BCR chain lengths, favoring formation of higher order oligomers (Fig. 2).

Tonic signaling from pre-BCR homo-interactions is apparently finely tuned to maintain remarkably low phosphorylation levels of receptors and associated signaling partners. Under optimal conditions, which include co-residency of receptors in domains, abundant levels of Syk and slower dimer off-rates, a sparse number (~100) of Syk molecules are engaging with pre-BCR at steady state in Nalm6 cells and perhaps one half of these are phosphorylated. This is consistent with the concept that autonomous signaling is judiciously regulated during early B cell development. There is a delicate balance since strong signaling can lead to apoptosis while failure of signaling prevents B cell progenitors from passing the critical pre-BCR checkpoint for B cell progenitor survival [43].

To put the general applicability of this model in context, one may regard our results as two applications of a mathematical model derived from general knowledge of the pre-B cell signaling system and calibrated to match two different cell lines. Since both samples were derived from leukemia patients, neither of them is necessarily typical of the normal functionality of pre-B cells. The applicability of our insights on the impact of changes in the receptor off-rate and of the membrane landscape on tonic signaling to other cell lines can potentially be verified by performing SPT studies like Erasmus [7].

Using single single-cell RNA-seq (scRNA-Seq) and proteomics, a recent paper has alluded to the existence of cellular heterogeneity present in the pre-BCR developmental pathway that could lead to distinct subsets of pre-BCR subtypes in B-cell acute lymphoblastic leukemia (B-ALL) [44]. A potential limitation in our model is that it assumes a homogenous population of cells and thus misses out on capturing biological heterogeneity based upon differential gene expression of pre-BCR signaling molecules. Further refinement of the model with this new information could lead to more a precise understanding of pre-BCR signaling dynamics.

Our work underscores the notion that patient-to-patient variablility may influence leukemia progression and dependency on pre-BCR signaling. Erasmus [7] noted that pre-BCR+ blasts from two B-ALL patients displayed diffusion characteristics that were similar either to 697 cells (patient# 238) or to Nalm6 cells (patient# 280). The slower overall diffusion rate for pre-BCR on Nalm6 cells and blasts from patient #280 are consistent with the modeling predictions that up to 75% of pre-BCR are engaged in dimerization or forming higher order oligomers at steady state. This translates to more efficient signaling, based upon higher levels of BCL6 expression, a downstream target of the pre-BCR signaling pathway.

Although the explanation for the differences in off-rates is not fully understood, we note that these two B-ALL groups can also be grouped based upon single nucleotide polymorphisms (SNPs) in the VpreB1 protein sequence that are predicted to alter the glycosylation of surrogate light chains in the pre-BCR [7]. We expect that continued exploration of variations in protein expression and receptor polymorphisms will generate a larger spectrum of cell-cell variability for pre-BCR signaling in the context of this form of leukemia.

The computational model presented here aims to describe the mechanism of tonic signaling through pre-B cell receptors on precursor (immature) B cells. Our model predictions are consistent with the observation by Erasmus et al [7] of increased receptor aggregation and Syk signaling on the Nalm6 cell line compared to the “697” cell line. Future experimental efforts similar to [7], applied to additional ALL derived cell lines, may help refine our models by including better estimations of receptor cluster sizes as well as receptor and kinase phosphorylation.

Tonic (ligand independent) signaling is also a feature of mature BCR, which is present in normal B cells and has also been linked to B cell malignancies [45]. However, mature B cell receptors are different from pre-BCR in that they are antigen-specific [46]. Furthermore, ligand independent BCR signaling has been associated with varied downstream signaling patterns, with potential feedbacks onto the signal initiation mechanism. We also note that recent work has associated BCR signaling with membrane microdomains [45], [47], [48]. This would motivate a possible future extension of our model to mature BCR signaling.

## 5 Conclusion

The spatial stochastic model of pre-BCR presented here was able to estimate a range of aggregate sizes and phosphorylation during autonomous signaling that is linked to variability among individuals. Our model explicitly simulates the two-dimensional movement and aggregation of pre-B cell receptors, their interaction with membrane bound Lyn and cytoplasmic Syk proteins and allows for transient co-confinement of pre-BCR within plasma membrane microdomains. The presence of microdomains impacts the pre-BCR chain lengths, favoring formation of higher order oligomers and increased signaling.

While the experimental data considered here was performed on leukemia blasts, cell-to-cell variability is likely to also be a distinguishing feature of normal progenitor B cells during B cell development, motivating additional studies to understand the threshold of pre-BCR signaling that is permissive for survival prior to light chain rearrangement. The latter represents the critical next step in B cell development, required for assembly of a functional BCR and its secretory immunoglobulin form. In the future, the spatial stochastic model can be exploited to evaluate the impact of additional variables on pre-BCR signaling and expanded to include additional downstream signaling components.

## Supplementary Material

supplemental

## Figures and Tables

**TABLE 1:** PRE-B Cell Receptor Cell Line Characteristics in the Model

	697	Nalm 6
No of receptors^a^	10000	10000
No of Lyn available to receptor^b^	10% of receptor	
No of Syk^a^	48290	274302
Binding radius (*μ*m)^c^	0.0001	
Dimer off rate (/s)^c^	1.14	0.159
Diffusion coefficient for monomeric receptors (*μ*m^2^/s)^d^	0.16	
Diffusion coefficient Lyn (*μ*m^2^/s)^e^	0.4	
Diffusion coefficient Syk (*μ*m^2^/s)^f^	17	

a Experimental data in this paper.

b Estimated from references [24], [25].

c,d Estimated from reference [7].

e Estimated from reference [26].

f Estimated from reference [27].

**TABLE 2:** LYN and SYK Molecule Binding Radii and Dimer Off Rate

Molecules	ITAM	Phos. Status	Binding radius (*μ*m)	Dimer off rate (/s)
Lyn	Ig*α*	0	2.29E-04^a^	20^b^
		≥ 1	2.29E-04^a^	0.12^b^
	Ig*β*	≥ 1	1.14E-04^c^	0.12^b^
Syk	Ig*α*	1	1.31E-04^d^	2.6^e^
		2	1.57E-03^d^	0.3^e^
	Ig*β*	1	4.37E-05^f^	2.6^e^
		2	5.25E-04^f^	0.3^e^

a,b Estimated from references [29], [16].

c Estimated according to observed experimental data (Lyn binding to Igβ is approximately 1/2 of the binding observed to Igα) in reference [28].

d Estimated from references [16], [30], [31].

e Estimated from reference [30].

f Estimated from references [30], [31], [32] where binding of Syk to Igα is 3x more than binding observed to Igβ; also reference [16].

**TABLE 3:** Kinase and Substrate Phosphorylation Status and Rates

Kinase	Phos. Status	Substrate	Phos. Status	Rate (/s)
Lyn	0	Ig*α*(Y182/Y193)	0	30^a^
	0		1	15^b^
	1	Ig*β*(Y195/206)	0	100^a^
	1		1	50^b^
	0	Lyn(Y397)	0	30^a^
	1			100^a^
	0	Syk(Y342/Y346)	0	30^a^
	1			100^a^
Syk	0	Syk(Y519/Y520)	0	100^a^
	1			200^a^

a See reference [29].

b Estimated from ref [13]. Phosphorylation of 2nd site on the ITAM occurs at half the rate from first.

**TABLE 4:** Dephosphorylation Rates

Substrate	Phos. Status	Rate (/s)
Ig*α*(Y182/Y193)	2	40^a^
	1	20^b^
Ig*β*(Y195/206)	2	40^a^
	1	20^b^
Lyn(Y397)	1	20^b^
Syk(Y342/Y346)		
Syk(Y519/Y520)		

a Estimated from references [29] and [13].Dephosphorylation of doubly phosphorylated ITAMs occur at 2x the rate of singly phosphorylated ITAM.

b Estimated from reference [29].
