# Supplementary material for: Spatial Stochastic Model of the Pre-B Cell Receptor
Source: IEEE/ACM Trans Comput Biol Bioinform. Author manuscript; Available in PMC 2023 Apr 24. (PMC10123485; doi:10.1109/TCBB.2022.3166149)
Supplement: supplemental [file NIHMS1871458-supplement-supplemental.docx]

Supplement

Kerketta et al.

Methods

The kinetic parameters of receptor-receptor binding and dissociation result from experimental data analysis and are the input for the detailed spatial simulations presented in this paper. Unlike larger scale models of cell signaling where $k_{\mathrm{on}}$ and $k_{\mathrm{off}}$ summarize the entirety of the molecular process, both the experimental procedure and the spatial simulations depend on additional details. As in our prior spatial stochastic models (1, 2), experimental observables that reflect receptor-receptor binding are both inputs of the simulation and provide reference for evaluating outputs of the simulation. To define unknown input parameters, we also employed a well-mixed stochastic simulation that represents a simplified version of the spatial model.

Overview of the parameter estimation workflow

Model validation by direct comparison to single particle tracking data requires simulation of the movement of individual receptors with a spatial and temporal resolution that matches or exceeds that of the experimental method. The simulation-experiment comparison raises issues of conversion between corresponding parameters. The discussion in Sections 1 – 3 in this Supplement provides details on our approach to the issue of extracting model and simulation parameters for receptor-receptor binding. Section 1 deals with the estimation of average on- and off-rates ($k_{\mathrm{on}},k_{\mathrm{off}}$) for individual receptor-receptor binding, based on pairs of bound particles as identified from two color single particle tracking (SPT). We developed a non-spatial agent-based simulation (NSAS) to provide the larger statistics required for this purpose. Related experimental considerations and the resulting estimates are presented in Section 2. The mapping of the non-spatial rate constant $k_{\mathrm{on}}$ for receptor-receptor binding to the spatial simulation is discussed in Section 3. The receptor-receptor binding radius was estimated by direct comparison of the predicted aggregate size distributions.

Sensitivity analysis

In Section 4 we briefly summarize the variability of important model outputs to changes in model parameters, obtained by performing dedicated simulations in the spatial model.

1. Using a well-mixed model to estimate on- and off-rates from two color labelled experimental data

1.1. Experimental setup as used for model building

Two-color labeling is used to identify bound receptor pairs.

Experimental data in this model are derived from single particle tracking of pre-BCR as described previously (3). In this protocol, a fraction of the receptors was labelled with red fluorescent Quantum dots (QDs) and another fraction with green fluorescent QDs. We assume the two fractions are the same,

$$f_{label}=\frac{N_{\mathrm{red}}}{N_{\mathrm{Rec}}}\approx\frac{N_{\mathrm{green}}}{N_{\mathrm{Rec}}}$$

and we estimate the labeling efficiency from the available data. Two-color QD labeling allows the imaging and separate identification of molecules that are separated by small distances, such as when they are bound in an oligomer. We assume that labeling does not change the properties of receptors.

The experimental procedure relies on movies, each comprised of sequences of images taken at a fixed time intervals (20 frames/sec). For each color, the procedure identifies trajectories of individual particles. A Hidden Markov Model (HMM) is used to identify pairs of bound receptors (comprised of 2 different QD colors), based on their mutual distance and movement patterns (4). Each dimer instance is identified by the two trajectories involved, and by the starting and final time point when the dimer was observed. The diffusion coefficient of dimers identified in this manner is reported separately from that of individual (monomer) trajectories. The mean and standard deviation of dimer lifetimes, as well as the diffusion coefficient of monomers are reported in the main text.

Dimers and monomers identified experimentally are not necessarily dimers and monomers.

Since the labeling efficiency is small (on the order of 1%), the majority of receptors are dark (do not carry any label and are not imaged). Consequently, when the two-color method is used on a system that can have monomer and dimer receptors, there is a significant chance that dimers are *not* identified as such. If one partner in a dimer is labelled, the probability that the other partner is labelled is equal to the labeling fraction, i.e. on the order of 1%. Thus, the majority of dimers that are imaged are likely identified as monomers. This needs to be accounted for when using the observed number of dimers and monomers to infer the relative dimer to monomer ratio.

In a system where higher aggregates are not possible, one can be confident that an observed dimer is in fact a dimer. There are indications that pre-B receptors form not only dimers, but larger oligomers. Importantly, an observed dimer can be assumed to be part of an oligomer that contains *at least* the two labelled receptors, but could be larger. Dimers (and higher oligomers) are not distinguished from monomers if they contain only one labelled receptor or if individual receptors in an aggregate are bound to the same color of quantum dot. Finally, since the HMM algorithm used to identify bound pairs typically uses a separation distance based upon structural features of a dimer (3, 4), it is possible that some larger oligomers escape detection in the automated analysis.

Experimental results used for modeling

Analysis was performed for two BCP-ALL cell lines (697 and Nalm6), based upon observations by Erasmus et al (3) that pre-BCR homointeractions in these two lines exhibit differential off-rates (3). Receptors on Nalm6 cells were observed to have higher observed dimer lifetimes; the monomer and dimer diffusion coefficients for Nalm6 cells were also were higher than for the 697 line. Finally, in rare cases, correlated movement was between QD-labelled receptor pairs with separations consistent with a dark receptor in the middle(3). All of this points to the possibility that Nalm6 has larger aggregates.

The experimental parameters estimated for the two lines are:

Dimer off-rate: $k_{\mathrm{off}}^{(697)}=1.14 s^{-1}, k_{\mathrm{off}}^{(Nalm6)}=0.23 s^{-1}$

Diffusion coefficient, monomers: $D_{\mathrm{mon}}^{(697)}=0.161\frac{\mu m}{s^{2}} , D_{\mathrm{mon}}^{(Nalm6)}=0.119\frac{\mu m}{s^{2}}$

Diffusion coefficient, dimers: $D_{\dim}^{(697)}=0.0591\frac{\mu m}{s^{2}} , D_{\mathrm{mon}}^{(Nalm6)}=0.0151\frac{\mu m}{s^{2}}$

In addition, we used the total observation time for dimers and monomers (the total aggregate length of trajectories identified) as well as measurements of the number of receptors per cell.

1.2. Well mixed simulations

We developed an agent-based*,* non-spatial stochastic simulation to estimate the parameters of receptor-receptor binding. These parameters were matched to the available experimental data and were the basis for the parameter set used in the spatial simulations reported in the paper.

Non-spatial agent-based simulation (NSAS)

The non-spatial simulation was written in Matlab. As in the spatial model, the program follows the state of each receptor, whether it is unbound or part of a dimer or larger complex. Note that we track individual receptors, bound or not. The term “aggregate” normally refers to a group of receptors that are bound to each other and diffuse as a single entity until they break apart.

Reactions are simulated event-by-event, similarly to the Gillespie SSA (5); at each update, system-wide propensities for each type of reaction are used to choose the time and type of the next event. Once the type of event is identified, an individual instance (molecule or molecule pair and binding site / bond) is selected, consistent with the state of the molecules in the system. Individual and system-wide propensities are re-calculated as necessary.

Reactions in the receptor-receptor binding sector consist of binding and dissociation. Each receptor has two receptor-binding sites, which can bind to available similar sites on any receptor that is not in the same aggregate (oligomer). There is no limit on the size of the resulting linear aggregates. Only receptors at the ends of an aggregate have free binding domains which can form a new bond. The main two parameters are the well-mixed on-rate $k_{\mathrm{on}}^{(\mathrm{sim})}$, defined as the rate (probability per unit time) for binding for a specific pair of monomers, in the simulation space (referred to as “per-pair” rate, $\left( \frac{\#\cdot s}{\mathrm{simsp}} \right)^{-1}$) and the intrinsic dimer off-rate $k_{\mathrm{off}}^{\left( \mathrm{sim} \right)} \left( \left( \#\cdot s \right)^{-1}\frac{s^{-1}}{\#} \right)$, defined as the rate for the dissociation of one specific receptor-receptor bond.

The likelihood of very large aggregates is limited by the way on- and off-rates depend on aggregate size. The on-rate for a pair of aggregates is a fraction of the monomer on-rate, consistent with the reduced mobility of oligomers. The diffusion coefficient is inversely proportional to the size of an aggregate; the on-rate for a pair of aggregates is proportional to the sum of their diffusion coefficients; for a pair of aggregates with $n$ and $m$ receptors we have $k_{\mathrm{on}}\left( m, n \right)=2\left( \frac{1}{n}+\frac{1}{m} \right)k_{\mathrm{on}}\left( 1,1 \right)$. The off-rate $k_{\mathrm{off}}^{\left( \mathrm{sim} \right)}$ is the same for all existing receptor-receptor bonds; an aggregate of size $m$ contains $m-1$ receptor-receptor bonds, thus a total dissociation rate $(m-1)k_{\mathrm{off}}^{\left( \mathrm{sim} \right)}$.

Events are generated randomly, based on the individual rates described above and the resulting propensities; association events result in the merging of two aggregates, and dissociation results in the separation of an aggregate into two smaller ones. The main output of the NSS receptor binding sector is a history of aggregates, their sizes, membership (identity of receptors they contain), and start and end times. This record is used to obtain dimer lifetimes and other quantities that can be compared directly to experimental data.

Simulated histories based upon 2-color quantum dot tracking

In order to compare with the experimentally observed dimer / monomer features, we have to emulate the 2-color QD labeling of receptors used for single particle tracking. This aspect is significant because the experimental identification of individual receptors requires a low labeling efficiency, typically $\lesssim1\%$; as a result, the vast majority of receptors are dark (not visible), and trajectories identified as monomers or dimers may belong to receptors that are part of dimers or higher aggregates, with dark partners.

Non-spatial (NSAS) simulations were performed for $N_{\mathrm{Rec}}=4000$ receptors, representing approximately half of the number of receptors in a pre-B cell; this corresponds to a setup where 80% of one side of a cell is imaged. At labeling fractions of $f_{\mathrm{label}}\approx0.25\cdots0.5\%,$ the model yields $10-20$trajectories of each color, resulting in a handful of dimer events per simulation run^[[1]](#footnote-1)^. Since one run of $t_{\mathrm{sim}}\approx600 s$ time takes on the order of an hour of clock time, it was impractical to perform a large number of NSAS simulations to obtain a good sample of simulated dimer events.

To increase the number of simulated histories that emulate the sparse 2-color experimental conditions, we employed a procedure of similar to repeated re-labeling. A simulated history is the complete list of binding and unbinding events in one NSAS run; each entry consists of the time, the nature of the event (binding or unbinding) and the identifier (ID number) or the receptors involved. Based on this, we can identify the time intervals over which a specific pair of receptor IDs were part of the same aggregate (bound either directly to each other or through one or more other receptors), as well as the time evolution of the size of the containing aggregate.

We randomly chose a subset of $f_{\mathrm{label}}\cdot N_{\mathrm{receptor}}$ receptors IDs for each of the two QD colors, and extracted the dimer instances for all red-green pairs, with their total duration and distribution of aggregate size. These “observed” dimer instances can be filtered based on the distance in the chain (number of intervening bonds) between the two labelled receptors. The resulting samples of simulated observable dimer lifetimes, dimer sizes (the size of the aggregate that contains the detectable dimer), as well as observable monomer sizes (the size of the aggregate containing a labelled receptor that is detected as a monomer), were collected for several passes of each NSAS simulation.

The collected dimer and monomer samples (typically for 100 simulations) were then used to obtain the distribution of observed dimer lifetimes and containing aggregate sizes for observed dimers and monomers. The observed dimer lifetimes were compared to those obtained experimentally. When trimers and higher oligomers are possible and can be identified as dimers, the observed dimer lifetime is typically smaller than the base (simulated) dimer dissociation rate, because a linear oligomer containing $m>2$ receptors has $m-1$ bonds, each of which have a rate of dissociation equal to that of a dimer^[[2]](#footnote-2)^.

The distribution of aggregate sizes in observed dimers and monomers could also be used to estimate the relative changes in the average diffusion coefficient between monomers and dimers in a cell line or between two cell lines.

2. Parameter estimation

2.1. Initial estimate for the on-rate parameter

The original pairwise on-rate of $k_{\mathrm{on}}^{(SIM)}=2.56\cdot{10}^{-4}\left( \frac{\#\cdot s}{\mathrm{simsp}} \right)^{-1}$ used in the NSAS simulation was derived from our previous work on the ErbB family of receptors (1, 2), which assumed a binding radius (BR) of $\sigma_{B}=3.1\cdot{10}^{-4}\mu m^{2}$ and a time step $\Delta t={10}^{-6}s$. Assuming the same binding radius for pre-BCR, we scaled the observed well mixed on-rate to account for the lower receptor density. The BCR diffusion coefficients were experimentally derived (3).

2.2. Deriving observed quantities and parameters from experimental data

Given a pair of on- and off-rates and a labeling fraction, the well mixed simulation can be used to obtain predictions for the steady state distribution of aggregate sizes. We used simulations of a system corresponding to $N_{\mathrm{Rec}}=4000$ receptors to derive observables that could be compared between the simulation output and experimental results, as follows.

Total recorded time

The experimental data consists of sets of recorded trajectories derived from individual cells, over $50 s$ of recording time, in two colors (red and green). These trajectories vary in length (time), and are typically shorter than the total recording time; this reflects various factors that impede imaging, including blinking, exit from the focal plane, or particle identification issues. We take the total time length of trajectories of one color ($T_{\mathrm{Red}}\approx T_{\mathrm{Green}}\approx T_{\mathrm{monomer}}$) as an overall measure of labeling efficiency, by comparing to the total expected number of receptors susceptible to be imaged in one such experiment.

We assume that the experimental process images 80% of one side of one individual cell; therefore, the total number of receptors that are available for imaging is $N_{\mathrm{Rec}}=4000\frac{\mathrm{rec}}{\mathrm{cell}}$ and, with perfect labeling, should result in $T_{\mathrm{total}}=T_{\mathrm{obs}}\cdot N_{\mathrm{Rec}}=50 sec\cdot4000\frac{\mathrm{rec}}{\mathrm{cell}}=2.0\cdot{10}^{5} \sec$ of recorded tracks of one color.

***Estimated labeling efficiency****.* For one cell line (697), the total time recorded for one QD color tracking in 215 cells was $101,960$ seconds in 7095 trajectories, an average of $T_{\mathrm{monomer}}\approx474 s$ per cell. The resulting labeling efficiency is $f_{\mathrm{label}}=\frac{474}{200,000}=0.237\%$. This estimation is approximately consistent with the assumed labeling efficiency of $f_{\mathrm{label}}=0.25\%$ used in the subsequent calculations. The total recorded time for one QD color tracking using the Nalm6 cell line was 13,958 sec.

*Considerations for comparing observed dimers.* The dimer identification criteria used for the two cell lines relied on different separation distance thresholds: for the 697 cell line we used $d_{\mathrm{sep}}^{\left( 697 \right)}=100 \mathrm{nm}$ corresponding to one receptor-receptor bond; for the Nalm6 cell line, this was increased to $d_{\mathrm{sep}}^{\left( 697 \right)}=300 \mathrm{nm}$, allowing up to three bonds intervening between the two receptors that were imaged. Thus, in simulations of the two cell lines aimed at comparisons with experimental observations from “697” cells, dimer events are restricted to a pair of adjacent red- and green-tagged receptors; for Nalm6 cells, the pair can be separated by up to three bonds or two additional receptors between them.

Observed dimer: monomer ratio and dimer lifetimes.

The observed dimer: monomer ratio is estimated as the total length (time) of trajectories identified as dimers, divided by the total length (time) of trajectories of *one* color in the same group of experiments.

For 697 cells, the total length of the 199 identified dimer trajectories was $T_{\mathrm{dimer}}^{(697)}=174.5 \sec$, resulting in a dimer to monomer ratio of $0.17\%$ or $T_{\mathrm{dimer}}^{\left( 697 \right)}/T_{\mathrm{monomer}}^{\left( 697 \right)} =1.7\times{10}^{-3}$, and the observed dimer lifetime was $\tau_{\mathrm{dimer}}^{(697)}=0.88 \sec$.

For Nalm6 cells, the total length of the 28 identified dimer trajectories was $T_{\mathrm{dimer}}^{(Nalm6)}=122.1 \sec$, resulting in an observed dimer to monomer ratio of $0.875\%$ or $T_{\mathrm{dimer}}^{\left( Nalm6 \right)}/T_{\mathrm{monomer}}^{\left( Nalm6 \right)} =8.75\times{10}^{-3} \sec$, and the observed dimer lifetime was $\tau_{\mathrm{dimer}}^{(Nalm6)}=4.35 \sec$.

The experimental information and the resulting estimates are summarized below.

|  | **697 cell line** | **Nalm6 cell line** |
| --- | --- | --- |
| Receptors per cell^#^ **(40% imaged) [#]** | 4000 | |
| Recording time (each cell) | 50 s | |
| Cell count [#] | 215 | 62 |
| Trajectory length (one color, total) | $1.02\times{10}^{5}$ s | $1.396\times{10}^{4}$ s |
| Maximum potential trajectory length^$^ | $4.3\times{10}^{7}$ s | $1.24\times{10}^{7}$ s |
| Estimated labeling efficiency* | $\mathbf{2}.\mathbf{37}\times\mathbf{10}^{-\mathbf{3}}$ | $\mathbf{1}.\mathbf{13}\times\mathbf{10}^{-\mathbf{3}}$ |
| Dimer event count (total)^&^ | 199^a^ | 28^b^ |
| Dimer trajectory length (total)^&^ | $174.5$s^a^ | $122.1$ s^b^ |
| Observed dimer: monomer ratio | $\mathbf{1}.\mathbf{71}\times\mathbf{10}^{-\mathbf{3}}$ | $\mathbf{8}.\mathbf{75}\times\mathbf{10}^{-\mathbf{3}}$ |
| Observed dimer lifetime | 0.88 s | 4.35 s |

**Notes:**

^#^Assuming 10,000 receptors per cell, imaging captures 80% of one side.

^$^If all receptors in the imaged portion of each cell could be recorded for 50 seconds.

^*^ We used a labeling efficiency of 0.25% in the non-spatial agent based simulations.

^&ab^ Dimers identified by HMM (Erasmus 2016) using different separation thresholds:

^a^ “697” cells: $d_{HMM}=100$ nm, corresponding to one intervening bond

^b^ Nalm6 cells: $d_{\mathrm{HMM}}=300$ nm, corresponding to three intervening bonds

2.3. On- and off-rates from well mixed simulations

For a given number of receptors and labeling fraction, the well mixed simulation (NSAS) has only two parameters, the (per-pair per simulation space) on-rate $k_{\mathrm{on}}^{(SIM)}$, and the off-rate $k_{\mathrm{off}}^{(SIM)}$. The on-rate represents the dimerization probability per time for one pair of receptor monomers enclosed in an area corresponding to the 4000 receptors used in the simulation. As discussed above, we used an initial estimate based on comparisons with previous simulations of ErbB2-ErbB3:

$$k_{\mathrm{on}}^{(SIM)}=2.5556\cdot{10}^{-4} \left( \frac{\#\cdot s}{\mathrm{simsp}} \right)^{-1}$$

Since time is the only dimensional quantity in the simulation, scaling all dimensional parameters with the same factor amounts to changing the unit of measure for the time; this will leave dimensionless quantities (such as ratios of time lengths) unchanged; thus, two simulations with the same $k_{\mathrm{on}}/k_{\mathrm{off}}$ ratio are equivalent.

We adopted the following strategy: We performed simulations with $N=4000$ receptors and a labeling fraction of $f=0.25\%$ (i.e. $N_{\mathrm{red}}=N_{\mathrm{green}}=10$ labeled receptors) for the same on-rate and a range of values for the off-rate; for each parameter set, we derived predictions for the dimer: monomer ratio (“DM ratio”) as well as for the predicted observed dimer lifetime. We used different thresholds for an “observable” dimer, 1 intervening bond for the 697 line, and up to 3 intervening bonds for the Nalm6 cell line.

To calibrate the model for 697 cells, we identified the off-rate that resulted in the observed DM ratio of $0.171\%$; this was achieved at $k_{\mathrm{off}}^{(SIM)}=1.760 s^{-1}$, corresponding to a dimer lifetime of $\tau_{D}^{SIM}=0.568$ s. We derived a scale factor to convert from time in the initial units of the simulation to physical units, consistent with the data we have for the 697 cell line, (i.e. $t^{\mathrm{phys}}=\varphi\cdot t^{SIM}$):

$$\varphi=\frac{\tau_{D}^{\left( 697 \right)}}{\tau_{D}^{SIM}}=\frac{0.88}{0.568}=1.5493\to k_{\mathrm{on}}^{\left( \mathrm{phys} \right)}=k_{\mathrm{on}}^{\left( \mathrm{SIM} \right)}\cdot\frac{1}{\varphi}=1.65\cdot{10}^{-4} \left( \frac{\#\cdot s}{\mathrm{simsp}} \right)^{-1}$$

The corresponding dimer off-rate in physical units is the same as the experimentally observed value for this cell line, namely $k_{\mathrm{on}}^{\left( 697 \right)}=\frac{1}{0.88}=1.136 s^{-1}$.

For the Nalm6 cell line, we used the same parameters as for the 697 line, with the exception of the dimer off-rate. Since in this case the observed dimers include higher aggregates where the separation between the labeled receptors is up to 3 bonds, the observed dimer lifetime is lower than the intrinsic value (the inverse of the dimer off-rate). We set the dimer off-rate to match the observed dimer lifetime of $\tau_{D,obs}^{(Nalm6)}=4.35 s$.

The resulting intrinsic dimer lifetime for Nalm6 is $\tau_{D}^{(Nalm6)}=6.28 s$ and the off-rate is $k_{\mathrm{off}}^{\left( Nalm6 \right)}=0.148 s^{-1}.$

3. From well mixed to spatial

The remaining issue is to map the well mixed on-rate to a binding radius (BR). A specific BR is associated with each binary reaction type. Consistent with our assumptions, we use the same BR for the association of two monomers and for the association of a pair of aggregates of any size. We set the BR in the spatial simulation by comparing the relative fractions of monomers, dimers, trimers, etc. with the well-mixed simulation calibrated for the 697 cell line (i.e. $k_{\mathrm{on}}^{\left( \mathrm{phys} \right)}=1.65\cdot{10}^{-4} \left( \frac{\#\cdot s}{\mathrm{simsp}} \right)^{-1}$ and $k_{\mathrm{off}}^{\left( 697 \right)}=1.136 s^{-1}$).

A.

B.

**Supplementary Figure 1**: Predictions from the well mixed (NSAS) simulation set used to calibrate simulation parameters; The plots represent predictions for (A) observed dimer: monomer ratio and (B) dimer lifetimes using the calibrated on-rate, as a function of the true dimer lifetime (the inverse of the dimer off-rate).

**Supplementary Figure 2**: The plots represent predictions for percent of receptors engaged as different sized aggregates. The simulations were run for a range of binding radii to match the well mixed 697 cell line predictions. No of receptors: 71. Dimer off rate used: 1.14/s.

4. Sensitivity analysis

We performed several additional simulations in order to assess the sensitivity of the simulation results to changes in the values of model parameters.

4.1 Sensitivity to the domain exit probability.

Supplementary Figure 3 shows the distribution of aggregate sizes in the spatial model with domains for the standard escape probability (0.2) and when the value is doubled (0.4) and halves (0.1). Also shown is the no-domain result (corresponding to escape probability 1).

**
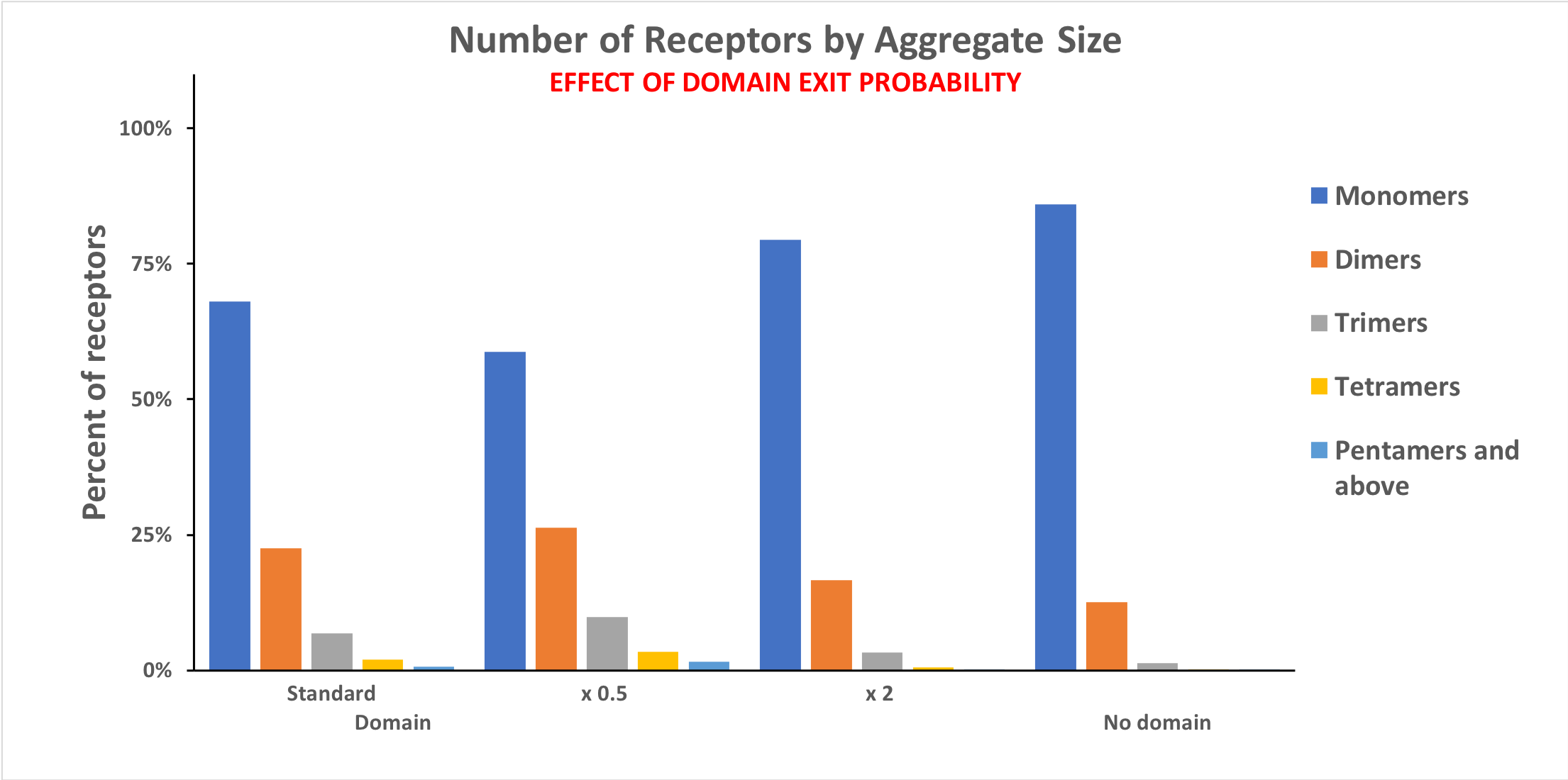
**

**Supplementary Figure 3:** The plots represent predictions in the full spatial model for percent of receptors engaged in different aggregate sizes in the domain model, with the standard escape probability, two modified values, and the no-domain model; the four sets correspond to escape probability values of 0.2, 0.1, 0.4, and 1.0.

**Supplementary Table 1:** Impact of domain exit probabilities (697 cell line)

Consistent with the discussion in the main document, increased domain trapping leads to higher aggregate sizes (and increased abundance of receptor-receptor bonds). This enhances receptor phosphorylation as well as the binding and phosphorylation of Lyn and Syk.

4.2 Sensitivity to Lyn kinetics

Results from simulations performed with modified values of parameters relating to the membrane bound Lyn protein are summarized in Supplementary Table 2. The effect of increased Lyn availability clearly results in increased binding and phosphorylation. Increased values of the Lyn diffusion coefficient and off-rates both result in reduced Lyn binding and receptor phosphorylation. Note that this cell line exhibits very low Lyn / Syk activation and binding; variations in Lyn phosphorylation and Syk binding likely reflect stochastic fluctuations rather than the effect of modified Lyn kinetics.

Supplementary Table 2. Sensitivity to modified Lyn kinetics

4.3 Sensitivity to the receptor-receptor bond strength (off rate)

The Nalm6 cell line was also simulated with two different off rates, in addition to the model value of 0.159 /s (consistent with the intersection in Supplementary Figure 1B), namely 0.230 /s (corresponding to the raw experimental off rate for this cell line) and 0.148 /s (corresponding to an estimate for the bond lifetime of 6.75 s). The resulting aggregate size distributions, Lyn / Syk binding, and phosphorylation are given in Supplementary Table 3.

Supplementary Table 3. Sensitivity to modified receptor-receptor off rates.

The effect of the ~7.5% decrease and ~27% increase in the dimer off rate is similar to that of the domain exit probability changes, in that the primary effect is an increase (respectively decrease) of the aggregate sizes. The further impact, on phosphorylation and binding of Lyn and Syk, is also consistent, in that lower off-rate (more stable receptor bonds) enhance signaling, and higher off-rates suppress it.

Supplemental References

1. Pryor, M. M., M. P. Steinkamp, A. M. Halasz, Y. Chen, S. Yang, M. S. Smith, G. Zahoransky-Kohalmi, M. Swift, X.-P. Xu, and D. Hanein. 2015. Orchestration of ErbB3 signaling through heterointeractions and homointeractions. *Molecular Biology of the Cell* 26: 4109-4123.

2. Kerketta, R., A. M. Halasz, M. P. Steinkamp, B. S. Wilson, and J. S. Edwards. 2016. Effect of Spatial Inhomogeneities on the Membrane Surface on Receptor Dimerization and Signal Initiation. *Front Cell Dev Biol* 4: 81.

3. Erasmus, M. F., K. Matlawska-Wasowska, I. Kinjyo, A. Mahajan, S. S. Winter, L. Xu, M. Horowitz, D. S. Lidke, and B. S. Wilson. 2016. Dynamic pre-BCR homodimers fine-tune autonomous survival signals in B cell precursor acute lymphoblastic leukemia. *Science Signaling* 9: ra116-ra116.

4. Low-Nam, S. T., K. A. Lidke, P. J. Cutler, R. C. Roovers, P. M. van Bergen en Henegouwen, B. S. Wilson, and D. S. Lidke. 2011. ErbB1 dimerization is promoted by domain co-confinement and stabilized by ligand binding. *Nat Struct Mol Biol* 18: 1244-1249.

5. Gillespie, D. T. 1977. Exact stochastic simulation of coupled chemical reactions. *J. Phys. Chem.* 81(5): 2340-23661.

1. The two-color experiments for single particle tracking require low labeling conditions. On average, we estimate that less than one in 100 dimer events could be observable even if all receptors were engaged in dimerization. This is a primary motivation for mathematical modeling. [↑](#footnote-ref-1)
2. The apparent dissociation rate is actually determined by the number $m^{'} (1\leq m^{'}\leq m)$ of intervening bonds between the observable receptors, so the dissociation rate is larger or equal to the true dimer off-rate. [↑](#footnote-ref-2)
